# Supplementary material for: Altered Transcription Factor Binding and Gene Bivalency in Islets of Intrauterine Growth Retarded Rats
Source: Cells. 2020 Jun 9;9(6):1435. doi: 10.3390/cells9061435 (PMC7348746; doi:10.3390/cells9061435)
Supplement: Supplementary file 1 [file cells-09-01435-s001.pdf]

**Supplemental Table S1 Percentage of the Distance from ChIP-Seq Peak to the Nearest T**

| Sample | 0-5 kbp (%) | 5-10 kbp (%) | 10-50 kbp (%) | 50-100 kbp (%) |
|---|---|---|---|---|
| H3K4me3-2wk-control | 16.89 | 8.40 | 23.22 | 14.68 |
| H3K4me3-2wk-IUGR | 36.50 | 7.74 | 24.75 | 11.82 |
| H3K4me3-10wk-control | 18.19 | 7.30 | 24.10 | 15.19 |
| H3K4me3-10wk-IUGR | 26.67 | 8.52 | 23.34 | 14.99 |
| H3K27me3-2wk-control | 15.03 | 8.28 | 30.85 | 15.94 |
| H3K27me3-2wk-IUGR | 38.06 | 11.07 | 17.91 | 10.08 |
| H3K27me3-10wk-control | 12.14 | 7.45 | 29.29 | 16.17 |
| H3K27me3-10wk-IUGR | 8.36 | 6.28 | 28.49 | 17.07 |
| H3K27Ac-2wk-control | 23.06 | 7.61 | 31.01 | 15.86 |
| H3K27Ac-2wk-IUGR | 27.75 | 8.14 | 31.24 | 14.27 |
| H3K27Ac-10wk-control | 19.18 | 9.85 | 35.07 | 15.55 |
| H3K27Ac-10wk-IUGR | 19.57 | 9.28 | 34.34 | 15.56 |

**ranscriptional Start Site**

| >100 kbp (%) |
|---|
| 36.82 |
| 19.19 |
| 35.21 |
| 26.48 |
| 29.90 |
| 22.87 |
| 34.94 |
| 39.81 |
| 22.47 |
| 18.60 |
| 20.36 |
| 21.25 |

**Supplemental Table S2 Number of Genes with Histone Mark Alterations in IUGR Islets**

| Histone Mark | Gene Number | |
|---|---|---|
| | **2 wk** | **10 wk** |
| **H3K4me3** | 2181 | 9988 |
| **H3K27me3** | 1482 | 3489 |
| **H3K27Ac** | 4572 | 4355 |

**Supplemental Table S3 Differentially Expressed Genes with Consistent H3K4me3 Histone Mark Cl**

**2-wk Islets**

| Gene | RNAseq_logFC | RNAseq_FDR | H3K4me3_avgpeak_logFC | H3K4me3_maxpeak_logFC |
|---|---|---|---|---|
| *Astl* | 4.752550028 | 0.03194251 | 3.64 | 3.64 |
| *Tlx2* | 2.469489609 | 0.00934619 | 2.65 | 2.65 |
| *Serpina3n* | 2.453790776 | 0.02156974 | 3.74 | 3.74 |
| *Gpd1* | 1.881588907 | 0.00959853 | 4.05 | 4.06 |
| *Osm* | 1.770787462 | 0.02244283 | 2.73 | 2.73 |
| *Cidec* | 1.618750565 | 0.04384976 | 2.6 | 2.6 |
| *Agt* | 1.262243743 | 0.01332097 | 3.38 | 3.38 |
| *Rgs16* | 1.056668238 | 0.04564087 | 1.5 | 3.41 |
| *Ccrl2* | 1.038984281 | 0.03404381 | 2.78 | 2.78 |
| *Nrarp* | 1.027764627 | 0.02890627 | 1.25 | 3.05 |
| *Nov* | 0.993259679 | 0.00222795 | 3.42 | 3.44 |
| *Anxa2* | 0.992594856 | 0.01197936 | 2.7 | 2.7 |
| *Map3k8* | 0.942927036 | 0.04353668 | 2.82 | 2.82 |
| *S100a4* | 0.939418931 | 0.02403073 | 2.64 | 2.64 |
| *Dgat2* | 0.914704136 | 0.01637179 | 2.22 | 2.22 |
| *Nedd9* | 0.892146789 | 0.00809417 | 4.26 | 4.27 |
| *Slc25a25* | 0.878995218 | 0.00814116 | 2.36 | 2.36 |
| *Rhob* | 0.845030768 | 0.02923193 | 3.28 | 3.28 |
| *Epha2* | 0.798677553 | 0.02856194 | 1.79 | 1.79 |
| *Cald1* | 0.702453809 | 0.00844655 | 2.69 | 2.69 |
| *Csrp2* | 0.663214473 | 0.01758449 | 3.63 | 3.63 |
| *Mgat3* | 0.657525452 | 0.01549967 | 3.05 | 3.05 |
| *Crip2* | 0.656159262 | 0.02323416 | 2.81 | 2.81 |
| *B4galt5* | 0.629756646 | 0.0255003 | 4.19 | 4.19 |
| *Cotl1* | 0.626300953 | 0.01586007 | 3.42 | 3.42 |
| *Arf2* | 0.615205081 | 0.04349908 | 2.82 | 2.82 |
| *Tagln2* | 0.58357451 | 0.02099762 | 2.88 | 2.88 |
| *Itsn1* | 0.541657129 | 0.04769307 | 3.43 | 3.43 |
| *Akap12* | 0.535926186 | 0.04349908 | 2.58 | 2.58 |
| *Cep95* | -0.505529663 | 0.03694043 | -0.91 | -0.91 |
| *Ccnt2* | -0.513860663 | 0.00973952 | -0.34 | -0.34 |
| *Rtel1* | -0.515567931 | 0.01637179 | -0.39 | -0.39 |
| *Tada2a* | -0.548507658 | 0.04114461 | -0.53 | -0.53 |
| *Safb2* | -0.610913907 | 0.01532851 | -0.5 | -0.5 |
| *Nprl3* | -0.630506909 | 0.0251378 | -0.45 | -0.45 |
| *Atg13* | -0.633234936 | 0.00885295 | -0.47 | -0.47 |
| *Akap8l* | -0.685436588 | 0.00030368 | -0.46 | -0.46 |
| *Eif4ebp2* | -0.801133571 | 0.00206344 | -0.34 | -0.34 |
| *B3galt2* | -1.377888338 | 0.00014334 | -0.85 | -0.85 |
| *Fam83f* | -1.45962118 | 3.69E-05 | -0.64 | -0.64 |
| *Gamt* | -1.814893853 | 0.0005379 | -0.41 | -0.41 |

**10-wk Islets**

| Gene | RNAseq_logFC | RNAseq_FDR | H3K4me3_avgpeak_logFC | H3K4me3_maxpeak_logFC |
|---|---|---|---|---|
| *Kcns1* | 3.019816271 | 0.02380284 | 1.92 | 1.92 |
| *Gpr143* | 2.730098427 | 0.03074334 | 0.56 | 0.57 |
| *RGD1559578* | 2.443421223 | 0.0261219 | 0.66 | 0.66 |
| *Synpr* | 2.428078477 | 0.03533339 | 2.31 | 2.32 |
| *Lhfpl5* | 2.002878365 | 1.51E-07 | 0.91 | 1.29 |
| *LOC499240* | 1.823970302 | 0.04602231 | 1.12 | 1.13 |
| *P2rx2* | 1.637683266 | 3.34E-05 | 0.72 | 0.72 |
| *Slc2a5* | 1.625874003 | 4.67E-06 | 0.9 | 1.1 |
| *Ssmem1* | 1.490087662 | 0.00863035 | 0.47 | 1.51 |
| *Chga* | 1.444320166 | 8.96E-06 | 1.88 | 1.88 |
| *Hs3st5* | 1.443737419 | 0.00834834 | 0.89 | 0.89 |
| *Mdga2* | 1.438560591 | 0.01219206 | 0.96 | 0.96 |
| *Trpa1* | 1.424274829 | 1.18E-05 | 0.53 | 0.53 |
| *Slc8a3* | 1.407423761 | 1.42E-05 | 0.52 | 0.52 |
| *Gucy2c* | 1.387149619 | 0.0193738 | 0.77 | 0.77 |
| *Necab2* | 1.357231133 | 0.00608167 | 1.73 | 1.73 |
| *Kcnip2* | 1.353693098 | 0.0100289 | 0.96 | 0.96 |
| *Hs3st6* | 1.113981102 | 0.04353871 | 0.75 | 1.27 |
| *Rab39a* | 1.109505166 | 0.00496314 | 2.6 | 2.6 |
| *B4galnt4* | 1.10336662 | 6.97E-06 | 0.8 | 0.8 |
| *Gpr85* | 1.091433728 | 0.02722762 | 1.31 | 1.31 |
| *Elovl4* | 1.068743197 | 0.0012388 | 0.69 | 0.69 |
| *Nrcam* | 1.064113276 | 5.37E-06 | 2.17 | 2.17 |
| *Sardh* | 1.041972691 | 0.00293517 | 0.54 | 1.32 |
| *Gng2* | 1.041550019 | 0.02106509 | 1.3 | 1.3 |
| *Ntm* | 1.032033408 | 0.00109163 | 1.7 | 1.77 |
| *Slc2a2* | 1.016290026 | 0.00918887 | 0.36 | 0.45 |
| *Syt4* | 1.002766096 | 0.00105843 | 0.85 | 0.85 |
| *Fbxo2* | 0.998585212 | 0.01142895 | 0.92 | 0.92 |
| *Gpr158* | 0.991507169 | 0.00015142 | 1.05 | 1.05 |
| *Hap1* | 0.987419561 | 0.0003022 | 0.59 | 0.59 |
| *Plcxd3* | 0.944351039 | 0.00078876 | 0.63 | 1.27 |
| *Lig4* | 0.943669037 | 8.11E-05 | 1.15 | 1.32 |
| *Scgn* | 0.935020013 | 0.00011851 | 0.74 | 0.88 |
| *Rfx6* | 0.931836759 | 5.49E-06 | 1.66 | 2.2 |
| *Slc35g2* | 0.928510566 | 0.00081256 | 0.72 | 0.89 |
| *Rpia* | 0.925880893 | 0.00028774 | 0.4 | 1.29 |
| *Tmsbl1* | 0.919423136 | 0.00185573 | 1.34 | 2.07 |
| *Nmnat2* | 0.916798059 | 0.0410414 | 1.84 | 1.84 |
| *Fkbpl* | 0.89733031 | 0.00451447 | 0.49 | 1.49 |
| *Pnpla3* | 0.893726968 | 0.01557725 | 0.27 | 1.11 |
| *Stard5* | 0.885345219 | 5.66E-06 | 0.32 | 1.09 |

| | | | | |
|---|---|---|---|---|
| *Slc3a1* | 0.872940179 | 0.00053906 | 1.86 | 1.86 |
| *Mtmr7* | 0.872169279 | 2.11E-05 | 1.44 | 1.44 |
| *Rexo4* | 0.861311475 | 8.87E-06 | 0.63 | 1.27 |
| *Gcgr* | 0.859937047 | 0.01876891 | 0.86 | 0.87 |
| *Hmgn3* | 0.857883021 | 0.00016493 | 0.89 | 0.89 |
| *Scg3* | 0.854003228 | 0.01025939 | 0.27 | 0.96 |
| *Gpr119* | 0.852347753 | 0.00353223 | 0.9 | 1.04 |
| *Tmem150c* | 0.850662811 | 0.00308922 | 2.37 | 2.37 |
| *Gria2* | 0.848613366 | 0.00190745 | 0.62 | 0.81 |
| *Pdzrn4* | 0.848384432 | 0.00290005 | 0.5 | 0.5 |
| *Nr1h4* | 0.847173777 | 0.00137156 | 1.22 | 1.22 |
| *Asic1* | 0.841823893 | 0.04300371 | 0.9 | 0.95 |
| *Bphl* | 0.83683459 | 0.00713735 | 0.6 | 0.6 |
| *Trpm5* | 0.830189818 | 0.02145042 | 1.27 | 1.27 |
| *Scg5* | 0.825205459 | 0.02309076 | 0.79 | 1.2 |
| *Ddx25* | 0.818666245 | 0.00602494 | 0.35 | 1.28 |
| *Sstr3* | 0.81699599 | 0.01510163 | 0.46 | 0.46 |
| *Syt13* | 0.816932762 | 0.00330779 | 0.83 | 0.98 |
| *Col16a1* | 0.812785853 | 0.0002585 | 1.3 | 1.71 |
| *Zfp612* | 0.811153362 | 2.80E-05 | 0.43 | 0.43 |
| *Ntrk2* | 0.809791613 | 7.79E-05 | 1.02 | 1.18 |
| *Kbtbd8* | 0.8089209 | 0.00428822 | 0.13 | 0.55 |
| *Dnai1* | 0.80808946 | 0.00755748 | 0.46 | 0.46 |
| *Rai2* | 0.807575174 | 0.01974744 | 0.95 | 1.32 |
| *Snap25* | 0.805578393 | 0.00176993 | 0.37 | 1.18 |
| *Ppfia2* | 0.803607367 | 0.014795 | 0.85 | 0.85 |
| *Tarsl2* | 0.796447871 | 0.00149469 | 0.77 | 1.23 |
| *Ffar3* | 0.795976653 | 0.00410102 | 1.06 | 1.06 |
| *Tmem63c* | 0.794489059 | 0.01584208 | 0.76 | 0.76 |
| *Gpr6* | 0.791913855 | 0.03789497 | 1.45 | 1.52 |
| *Cyp4f17* | 0.791037331 | 0.04119738 | 0.33 | 1.11 |
| *Arc* | 0.788418732 | 0.03891885 | 0.73 | 1.22 |
| *Car8* | 0.788033106 | 0.00462783 | 0.07 | 0.98 |
| *Nup37* | 0.784155875 | 0.00018392 | 1.56 | 1.57 |
| *Ppm1e* | 0.783966628 | 0.00624413 | 1.65 | 1.65 |
| *Enah* | 0.781921388 | 0.00023424 | 1.27 | 1.27 |
| *Prnp* | 0.781883459 | 0.01975791 | 1 | 1.21 |
| *Vsnl1* | 0.778821051 | 0.0020343 | 0.95 | 0.95 |
| *Prlr* | 0.776191043 | 0.00085836 | 1 | 1.3 |
| *Gabrb3* | 0.775028723 | 0.02621619 | 1.22 | 1.22 |
| *Pgbd5* | 0.769436896 | 0.00919409 | 1.27 | 1.27 |
| *Nefh* | 0.768497747 | 0.01496554 | 0.69 | 0.69 |
| *Scn3b* | 0.768411634 | 0.00415148 | 0.75 | 0.91 |
| *Dnajc6* | 0.76800331 | 0.00398193 | 0.73 | 0.73 |
| *Slc8a1* | 0.765730644 | 0.00636317 | 1.47 | 1.47 |

| | | | | |
|---|---|---|---|---|
| *Peg3* | 0.761570075 | 0.02757085 | 0.91 | 1.27 |
| *Zcchc12* | 0.756034854 | 0.00611011 | 1.24 | 1.61 |
| *Abat* | 0.752327289 | 0.00603307 | 1.09 | 2.36 |
| *Myt1l* | 0.750484629 | 0.01818211 | 0.43 | 1.09 |
| *Ppp1r14c* | 0.74599674 | 0.00474445 | 0.86 | 0.86 |
| *LOC10254884* | 0.745914276 | 0.00877344 | 0.77 | 0.94 |
| *Pdia6* | 0.745799499 | 0.03809523 | 0.16 | 0.82 |
| *Col27a1* | 0.744514839 | 0.00473253 | 1.16 | 1.37 |
| *Zfp157* | 0.744062005 | 6.42E-05 | 0.04 | 1.02 |
| *Gstz1* | 0.742741477 | 0.00227633 | 0.07 | 1.06 |
| *Bicd1* | 0.738305961 | 0.00044739 | 1.51 | 1.51 |
| *Alg2* | 0.73646792 | 0.00039618 | -0.02 | 0.67 |
| *Ociad2* | 0.735493678 | 0.00124078 | 1.06 | 1.26 |
| *Neurod1* | 0.733179257 | 0.00446342 | 0.43 | 0.43 |
| *Mir3547* | 0.730776271 | 0.04371102 | 0.6 | 1.3 |
| *Giot1* | 0.724438695 | 0.01074649 | 0.53 | 0.53 |
| *Abcc3* | 0.721813222 | 0.00128267 | 0.68 | 0.68 |
| *Atp1b1* | 0.720103837 | 0.00083665 | 0.83 | 0.83 |
| *Hmgcll1* | 0.717957658 | 0.02302681 | 0.64 | 0.64 |
| *Slc38a4* | 0.715888924 | 0.00415122 | 1.62 | 1.62 |
| *Papss2* | 0.715183291 | 0.00116724 | 0.78 | 0.78 |
| *Hadh* | 0.707144072 | 0.01202588 | 0.81 | 0.98 |
| *Zfp329* | 0.70657005 | 0.00021238 | 0.82 | 0.82 |
| *Ccdc126* | 0.70558542 | 0.00941865 | 0.29 | 0.95 |
| *Tnik* | 0.70436911 | 0.00337105 | 0.63 | 0.63 |
| *Ostc* | 0.697938034 | 0.01829226 | -0.05 | 0.8 |
| *Limch1* | 0.697016217 | 0.02394906 | 0.49 | 0.49 |
| *Hyou1* | 0.696127053 | 0.04614924 | 0.73 | 0.87 |
| *Ryr2* | 0.694624975 | 0.01363057 | 0.92 | 0.92 |
| *Maged2* | 0.693508248 | 0.00059647 | 0.79 | 0.79 |
| *Pdx1* | 0.692310523 | 0.0128299 | 0.09 | 1.19 |
| *Rnf130* | 0.691911669 | 0.00245356 | 1.43 | 1.61 |
| *Ucn3* | 0.691361396 | 0.01868868 | 1.54 | 2.33 |
| *Camk1g* | 0.689385202 | 0.02999237 | 1.58 | 2.47 |
| *Tmem98* | 0.687419682 | 0.0008555 | 1.27 | 2.2 |
| *Comt* | 0.686553757 | 0.00346264 | 0.44 | 1.38 |
| *Pcdh9* | 0.685965596 | 0.00239237 | 0.64 | 0.64 |
| *Kcnh6* | 0.681420675 | 0.0047098 | 1.18 | 1.31 |
| *Ntan1* | 0.679783858 | 0.00058467 | 0.48 | 1.21 |
| *Pdia4* | 0.678174545 | 0.01485702 | 0.1 | 0.7 |
| *Bex2* | 0.677793719 | 0.00506114 | 0.71 | 0.71 |
| *Yipf6* | 0.677641815 | 0.0032535 | 0.13 | 0.93 |
| *Tnfrsf11b* | 0.677109165 | 0.0193738 | 1.14 | 1.15 |
| *Thnsl1* | 0.673281931 | 0.01360338 | 0.17 | 0.56 |
| *Pus3* | 0.672497812 | 0.00584538 | 0.09 | 1.28 |

| | | | | |
|---|---|---|---|---|
| *RGD1309534* | 0.671783942 | 0.00418484 | 0.55 | 0.55 |
| *Suox* | 0.671224244 | 0.00604971 | 0.13 | 0.89 |
| *Me1* | 0.67032375 | 0.00408819 | 1.28 | 1.28 |
| *Nipsnap1* | 0.670093656 | 0.00186353 | 0.64 | 0.64 |
| *Filip1* | 0.669956816 | 0.00360355 | 0.56 | 0.57 |
| *Eva1a* | 0.667277971 | 0.04976736 | 0.64 | 1.74 |
| *Usp51* | 0.667063978 | 0.04620409 | 0.31 | 1.03 |
| *Lrp11* | 0.664763561 | 0.01558795 | 1.05 | 1.05 |
| *Rufy3* | 0.663495072 | 0.00100163 | 0.54 | 0.6 |
| *Gck* | 0.659754431 | 0.01807655 | 1.19 | 1.4 |
| *Zfp955a* | 0.654256161 | 0.00213099 | 1.52 | 1.52 |
| *Scn2b* | 0.647775867 | 0.04260911 | 1.33 | 1.77 |
| *Epb41l3* | 0.644789316 | 0.00329012 | 0.43 | 1.24 |
| *Nkiras1* | 0.640789691 | 0.00791521 | 0.64 | 1.28 |
| *Tm4sf4* | 0.639564498 | 0.00214257 | 1.01 | 1.01 |
| *Cmbl* | 0.638627697 | 0.0056656 | 1.04 | 1.04 |
| *Rbmx2* | 0.638406507 | 0.02169134 | 0.99 | 0.99 |
| *Chrnb2* | 0.636265152 | 0.04822136 | 1.39 | 1.52 |
| *Eri2* | 0.635861137 | 0.00790825 | 0.11 | 1.09 |
| *Map2* | 0.63489457 | 0.00895403 | 1.48 | 1.48 |
| *Slc25a14* | 0.634645678 | 0.03007938 | -0.1 | 0.7 |
| *Dnaja4* | 0.634595617 | 0.0187605 | 0 | 0.97 |
| *Sdf2l1* | 0.633786059 | 0.03214424 | 0.16 | 0.78 |
| *Mrpl49* | 0.632608035 | 0.00402748 | 0.24 | 1.45 |
| *Ndrg4* | 0.632373711 | 0.0497661 | 1.67 | 1.68 |
| *Nme3* | 0.629972319 | 0.00319567 | 0.59 | 1.54 |
| *Tmed7* | 0.628550189 | 0.0077057 | 0.35 | 1.25 |
| *Dnpep* | 0.627657821 | 0.00304082 | 0.62 | 1.83 |
| *Sec23b* | 0.626866683 | 0.02257477 | 0 | 0.74 |
| *Snrpn* | 0.625839526 | 0.00079453 | 0.12 | 0.71 |
| *Glb1l2* | 0.625027707 | 0.00343375 | 1.5 | 1.5 |
| *Unc13a* | 0.618663763 | 0.01013492 | 0.77 | 1.01 |
| *Kcnj11* | 0.617885705 | 0.03652359 | 1.38 | 1.71 |
| *Ankrd33b* | 0.617149191 | 0.04367622 | 0.71 | 1.87 |
| *Tmem41b* | 0.617059944 | 0.0025354 | 1.3 | 1.3 |
| *Gnptg* | 0.616647545 | 0.00357204 | 0.26 | 1.1 |
| *Zfr* | 0.615838167 | 0.00155164 | 0.38 | 2.05 |
| *Arap2* | 0.614783225 | 0.00054923 | 0.77 | 0.77 |
| *Cmas* | 0.614696229 | 0.00345708 | 0.47 | 1.03 |
| *Pex5l* | 0.613982848 | 0.04125852 | 1.12 | 1.13 |
| *Tssc4* | 0.613696367 | 0.00807301 | 0.73 | 1.94 |
| *Mrps33* | 0.612314249 | 0.01051065 | 0.05 | 0.29 |
| *Surf2* | 0.612047238 | 0.00076941 | 0.69 | 1.2 |
| *Atpif1* | 0.611500684 | 0.00148021 | 0.14 | 0.52 |
| *Nell1* | 0.611448697 | 0.04383672 | 1.27 | 1.27 |

| | | | | |
|---|---|---|---|---|
| *Scn3a* | 0.606808001 | 0.02830647 | 0.94 | 0.94 |
| *Pde4dip* | 0.604588327 | 0.00362466 | 0.89 | 0.89 |
| *Camk2b* | 0.603192583 | 0.04436905 | 0.84 | 1.04 |
| *Agfg1* | 0.601817116 | 0.00139836 | 0.48 | 1.26 |
| *Ric8b* | 0.599721841 | 0.00101271 | 0.12 | 0.37 |
| *Pcdha8* | 0.599629915 | 0.04026407 | 1.22 | 1.22 |
| *Cep70* | 0.599547839 | 0.0144604 | 2.34 | 2.34 |
| *Fam175b* | 0.598521016 | 0.00069961 | 0.02 | 1.05 |
| *Manba* | 0.597386247 | 0.0024217 | 0.53 | 0.53 |
| *Gdpd1* | 0.597255627 | 0.0224421 | 0.3 | 1.09 |
| *Slc37a4* | 0.597091722 | 0.00830248 | 0.81 | 1.03 |
| *Mpi* | 0.596504066 | 0.01152248 | 0.7 | 1.63 |
| *Bag2* | 0.596088293 | 0.02630077 | 1.23 | 1.23 |
| *Gjd2* | 0.595979788 | 0.03360501 | 1.07 | 1.07 |
| *Btg3* | 0.595866886 | 0.00537311 | 0.15 | 0.8 |
| *Gramd3* | 0.594462685 | 0.00310603 | 1.2 | 1.2 |
| *Kif12* | 0.593607236 | 0.01315053 | 0.9 | 0.9 |
| *Tma7* | 0.593164226 | 0.00880317 | 0.62 | 1.67 |
| *Tmem38a* | 0.591181948 | 0.02340908 | 0.5 | 0.96 |
| *Scfd1* | 0.590420153 | 0.01624514 | 0.7 | 0.7 |
| *Rab40b* | 0.588373633 | 0.02160123 | 0.62 | 1.13 |
| *Cxxc4* | 0.58684389 | 0.01371722 | 0.18 | 0.79 |
| *Ppp1r1a* | 0.586541529 | 0.04961413 | 0.87 | 0.87 |
| *Gprasp1* | 0.585639248 | 0.030709 | 0.86 | 1.39 |
| *Ctsf* | 0.584819135 | 0.01069242 | 1.5 | 1.5 |
| *Manf* | 0.583528742 | 0.02899106 | 0.01 | 0.75 |
| *Slc25a20* | 0.582351931 | 0.00565709 | 0.47 | 0.47 |
| *Ffar1* | 0.578227904 | 0.04997614 | 0.86 | 1.06 |
| *Pc* | 0.577166468 | 0.00458742 | 0.45 | 1.29 |
| *Ahi1* | 0.577161717 | 0.00596101 | 1.44 | 1.85 |
| *Rundc3a* | 0.576693672 | 0.04872477 | 0.48 | 0.48 |
| *Sgcb* | 0.575664028 | 0.00734342 | 0.85 | 0.85 |
| *Naa20* | 0.575037064 | 0.00435629 | 0.23 | 0.65 |
| *Ddc* | 0.569889536 | 0.04934185 | 0.58 | 1.49 |
| *RGD1566386* | 0.565379395 | 0.01900664 | 1.25 | 1.25 |
| *Plekha8* | 0.563698161 | 0.00184079 | 0.26 | 1.51 |
| *Vegfa* | 0.562409983 | 0.02351337 | 0.29 | 1.23 |
| *Fbxo44* | 0.562134852 | 0.03713837 | 0.92 | 0.92 |
| *Ogdhl* | 0.558480466 | 0.03363744 | 0.83 | 0.83 |
| *Atp6v0e2* | 0.558255482 | 0.00413979 | 1.26 | 1.26 |
| *Fam135a* | 0.557871626 | 0.00487837 | 1.29 | 1.55 |
| *Sar1b* | 0.556760867 | 0.01444288 | 0.6 | 0.6 |
| *C1d* | 0.55611488 | 0.0105193 | 0.54 | 1.23 |
| *Tgoln2* | 0.555297084 | 0.03037216 | 0.16 | 1.07 |
| *Eno2* | 0.554156069 | 0.03203869 | 0.49 | 0.58 |

| | | | | |
|---|---|---|---|---|
| *Usp14* | 0.551893632 | 0.00532668 | 1.37 | 1.74 |
| *Cisd2* | 0.551048078 | 0.00317471 | 0.36 | 0.86 |
| *Ube2v2* | 0.550777148 | 0.00358389 | 0.43 | 0.43 |
| *Spint1* | 0.548647095 | 0.00317451 | 0.89 | 2.39 |
| *Thumpd3* | 0.547413217 | 0.01720214 | 0.38 | 0.38 |
| *Tlcd1* | 0.546402476 | 0.04874411 | 0.85 | 1.12 |
| *Aacs* | 0.544221994 | 0.01389723 | 1.13 | 1.9 |
| *Zfp14* | 0.54180731 | 0.02935818 | 0.14 | -0.84 |
| *Tmed4* | 0.540979668 | 0.00710081 | 0.5 | 0.5 |
| *Slc30a9* | 0.539943885 | 0.00638252 | 1.5 | 1.5 |
| *Mob4* | 0.53715804 | 0.01146981 | 0.37 | 0.37 |
| *Ptgr2* | 0.536582217 | 0.01146008 | 1.3 | 1.3 |
| *Lrrc39* | 0.536081214 | 0.03912598 | 2.02 | 2.02 |
| *Cacnb2* | 0.535145019 | 0.02256752 | 0.52 | 0.52 |
| *Zfp68* | 0.533147262 | 0.01014502 | 0.23 | 0.53 |
| *Golga5* | 0.533013498 | 0.01581617 | 0.06 | 0.57 |
| *Snx14* | 0.53286101 | 0.00452542 | 0.39 | 1.29 |
| *Trappc1* | 0.530093701 | 0.01149717 | 0.4 | 0.4 |
| *Arfip1* | 0.53000713 | 0.00873741 | 0.12 | 0.25 |
| *Tspyl2* | 0.526694444 | 0.01292347 | 0.59 | 0.99 |
| *RGD1311899* | 0.526487513 | 0.01421144 | 0.13 | 1.11 |
| *Prosc* | 0.525121747 | 0.02688618 | 0.47 | 0.95 |
| *Eif1a* | 0.523210676 | 0.01531451 | 0.35 | 1.25 |
| *Fam110b* | 0.522498479 | 0.02830647 | 0.58 | 1.34 |
| *Rab3a* | 0.51589525 | 0.0324379 | 0.34 | 0.78 |
| *Ahcyl2* | 0.515521981 | 0.01193697 | 0.33 | 0.33 |
| *Ddx41* | 0.51061324 | 0.01785673 | 1.77 | 2.01 |
| *Mrpl53* | 0.510419197 | 0.02626997 | 0.21 | 0.95 |
| *Cttn* | 0.509909451 | 0.01240167 | 0.49 | 0.54 |
| *MGC94199* | 0.508772094 | 0.02247027 | 0.14 | 1.65 |
| *B4galt4* | 0.505442045 | 0.04889539 | 0.97 | 0.97 |
| *Actr10* | 0.504774618 | 0.01849615 | 1.65 | 1.65 |
| *Ghr* | 0.503651457 | 0.03948713 | 2.02 | 2.03 |
| *Pld3* | 0.503574592 | 0.02033336 | 0.78 | 1.95 |
| *Yme1l1* | 0.50268064 | 0.0333998 | 0.95 | 0.95 |
| *Tfam* | 0.501433582 | 0.01479307 | 0.9 | 2 |
| *Tsr3* | 0.501282991 | 0.04635035 | 0.26 | 1.1 |
| *Zkscan1* | 0.501017403 | 0.01398216 | 0.81 | 0.94 |
| *Slc31a2* | 0.500034524 | 0.04924772 | 0.54 | 1.4 |
| *Prps1* | 0.497104596 | 0.01501762 | 0.02 | 0.02 |
| *Mthfs* | 0.496909673 | 0.02302681 | 0.41 | 1.39 |
| *Slc30a5* | 0.495730547 | 0.02945868 | 1.13 | 2.22 |
| *Rhbdd3* | 0.495709649 | 0.04994089 | 0.96 | 1.14 |
| *Soat1* | 0.495281888 | 0.02279531 | 1.56 | 1.57 |
| *St6gal1* | 0.495043536 | 0.03588131 | 0.72 | 0.72 |

| | | | | |
|---|---|---|---|---|
| *Plekhh1* | 0.494475981 | 0.03977416 | 1.12 | 1.13 |
| *Rab1b* | 0.493738761 | 0.00805212 | 0.5 | 1.5 |
| *Rogdi* | 0.493700416 | 0.00907454 | 0.64 | 1.03 |
| *Cops3* | 0.4936722 | 0.01466965 | 0.27 | 0.66 |
| *Tmem181* | 0.493393877 | 0.02325792 | 0.9 | 1.54 |
| *Agpat4* | 0.492806027 | 0.01168918 | 1.34 | 1.34 |
| *Arl14ep* | 0.490651839 | 0.01693837 | 0.91 | 0.91 |
| *Mrpl47* | 0.489446001 | 0.04847642 | 0.08 | 0.08 |
| *Fn3krp* | 0.487385482 | 0.01485484 | 0.11 | 0.11 |
| *Pxylp1* | 0.483897847 | 0.02489014 | 0.29 | 1.57 |
| *Hmgn1* | 0.483371269 | 0.0302896 | 0.96 | 1.2 |
| *Camlg* | 0.480599702 | 0.03961488 | 1.01 | 1.01 |
| *Dut* | 0.480573709 | 0.01834356 | -0.12 | 0.69 |
| *Armcx1* | 0.479015987 | 0.02516752 | 0.82 | 0.89 |
| *Gdi1* | 0.47817408 | 0.02516928 | 0.53 | 0.53 |
| *Prkar1a* | 0.476901615 | 0.0410414 | 0.43 | 1.27 |
| *LOC1001253[illegible]* | 0.474743789 | 0.03393972 | 0.41 | 1.14 |
| *Dnajc27* | 0.473529262 | 0.0327259 | 2.64 | 2.64 |
| *Tgds* | 0.472976312 | 0.02506803 | -0.02 | 0.82 |
| *Atxn7l3b* | 0.472489487 | 0.03781411 | 1 | 1.53 |
| *Maoa* | 0.47050644 | 0.03331502 | 0.26 | 0.76 |
| *Asah1* | 0.469672681 | 0.03396349 | 0.47 | 1.01 |
| *Atp6v0e1* | 0.467802828 | 0.01907711 | 0.42 | 1.02 |
| *Nme7* | 0.467027544 | 0.02870585 | 0.72 | 1.64 |
| *Mcf2l* | 0.466933434 | 0.05002442 | 1.17 | 1.57 |
| *Zfp26* | 0.466441906 | 0.00870709 | -0.15 | 0.59 |
| *Suco* | 0.463490487 | 0.01560548 | 0.69 | 0.69 |
| *Necap1* | 0.459818413 | 0.03886964 | 1.11 | 1.11 |
| *Trim23* | 0.456834295 | 0.04960148 | 1.29 | 1.29 |
| *Cstf2t* | 0.45407424 | 0.0151486 | 0.72 | 0.72 |
| *Vezt* | 0.452866767 | 0.01914468 | 0.63 | 1.55 |
| *Unc79* | 0.452571201 | 0.02848338 | 1.21 | 1.21 |
| *Erc1* | 0.451699648 | 0.03908514 | 0.08 | 0.44 |
| *Bre* | 0.450052451 | 0.02105566 | 0.23 | 1.8 |
| *Vsig10* | 0.448291746 | 0.0337337 | 0.72 | 0.81 |
| *Tsr2* | 0.448253399 | 0.03220466 | 0.19 | 0.19 |
| *Klhl9* | 0.44723909 | 0.03910276 | 0.12 | 0.62 |
| *Ttc9c* | 0.446609329 | 0.0400227 | 1.09 | 1.58 |
| *Ap3m1* | 0.445471378 | 0.04960148 | 0.25 | 1.67 |
| *G3bp2* | 0.442830414 | 0.02289787 | 0.57 | 1.16 |
| *Msmo1* | 0.442041732 | 0.03693488 | 1.19 | 1.19 |
| *RGD1310352* | 0.440924904 | 0.04165973 | 0.03 | 0.63 |
| *Fem1b* | 0.440599893 | 0.04193872 | 0.64 | 0.64 |
| *Slc35f5* | 0.440526578 | 0.04238203 | 0.1 | 0.94 |
| *Dhx36* | 0.440329001 | 0.02100277 | -0.13 | 0.57 |

| | | | | |
|---|---|---|---|---|
| *Zranb2* | 0.43980624 | 0.04103966 | 0.15 | 0.77 |
| *Vamp8* | 0.437378203 | 0.04440818 | 0.85 | 0.85 |
| *Entpd4* | 0.437144519 | 0.02865639 | 0.54 | 0.54 |
| *Ufd1l* | 0.435899897 | 0.03066224 | 0.14 | 0.52 |
| *Cep83* | 0.434526479 | 0.04094042 | 0.19 | 0.95 |
| *Gosr2* | 0.434327462 | 0.04630527 | 0.68 | 1.35 |
| *Dalrd3* | 0.433394099 | 0.03275101 | 0.39 | 0.77 |
| *Btbd3* | 0.433098237 | 0.03140804 | 1.66 | 1.66 |
| *Fbxo3* | 0.430137638 | 0.04003699 | 0.32 | 1.01 |
| *Stx16* | 0.429949618 | 0.03423596 | 0.99 | 1.69 |
| *Ttc21b* | 0.428897573 | 0.04888601 | 0.06 | 0.06 |
| *Bin1* | 0.428838781 | 0.03425842 | 0.87 | 1.21 |
| *Trappc13* | 0.42883199 | 0.03982835 | 1.29 | 1.29 |
| *Chkb* | 0.428771623 | 0.03730886 | 0.63 | 1.79 |
| *Acbd3* | 0.428733889 | 0.02764968 | 0.2 | 0.78 |
| *Hcfc2* | 0.428086294 | 0.04997614 | 1.24 | 1.24 |
| *Tomm20* | 0.427956637 | 0.04800199 | 0.11 | 0.11 |
| *Prpf3* | 0.427869298 | 0.02520513 | 0.77 | 0.93 |
| *Gtf3c3* | 0.42479805 | 0.02923666 | -0.13 | 1.28 |
| *Zfp386* | 0.424791805 | 0.04921037 | 0.46 | 1.31 |
| *Araf* | 0.421860083 | 0.04890163 | 0.57 | 0.58 |
| *Rad17* | 0.420799997 | 0.04633325 | 0.16 | 0.16 |
| *Lrrc8d* | 0.417525849 | 0.03574673 | 1.01 | 1.27 |
| *Srpk2* | 0.417507242 | 0.02292305 | 1.05 | 1.05 |
| *Atf2* | 0.416532631 | 0.04145704 | 0.02 | 0.71 |
| *Rab3gap2* | 0.414542756 | 0.0284655 | 0.37 | 0.37 |
| *Rab11a* | 0.414021798 | 0.03498503 | 0.13 | 1.13 |
| *Tmem57* | 0.407916477 | 0.03810934 | 0.06 | 0.06 |
| *Cdk5rap2* | 0.401702161 | 0.04094042 | 0.03 | 0.84 |
| *Lrig2* | 0.387656927 | 0.04251052 | 0.38 | 2.01 |
| *Cdc27* | 0.381754315 | 0.04913524 | 0.32 | 0.86 |
| *Rsbn1* | 0.361020106 | 0.04892808 | 0.2 | 1.4 |
| *Camk2d* | -0.413516362 | 0.04335305 | -0.05 | -0.1 |
| *Sft2d2* | -0.450648429 | 0.03783181 | -0.33 | -0.33 |
| *Jup* | -0.457002166 | 0.02033336 | -0.02 | -2.13 |
| *Man1c1* | -0.45725709 | 0.04976547 | -0.59 | -0.59 |
| *Efhd2* | -0.463030626 | 0.04110173 | -0.15 | -0.15 |
| *Mast2* | -0.466273587 | 0.03983825 | -0.19 | -0.86 |
| *Dgkz* | -0.479568925 | 0.03657736 | -0.47 | -0.47 |
| *Dusp6* | -0.482828618 | 0.04605076 | -0.73 | -0.73 |
| *Eif3h* | -0.49051131 | 0.03769312 | -0.51 | -0.51 |
| *Htra2* | -0.49468217 | 0.03740255 | -0.51 | -0.51 |
| *Rsu1* | -0.505390166 | 0.04302107 | -0.4 | -0.4 |
| *Myl12a* | -0.505895528 | 0.02183961 | -0.68 | -0.68 |
| *Map3k5* | -0.516730476 | 0.02158263 | -0.51 | -0.55 |

| | | | | |
|---|---|---|---|---|
| *Arhgap21* | -0.520551542 | 0.02425255 | -0.25 | -0.25 |
| *Slc35e3* | -0.536903401 | 0.04980604 | -0.85 | -0.85 |
| *Wwp1* | -0.544378917 | 0.0076127 | -0.53 | -0.53 |
| *Klhl21* | -0.545235313 | 0.03297647 | -0.63 | -0.63 |
| *Rxra* | -0.553848639 | 0.0121195 | -0.27 | -0.27 |
| *Cracr2b* | -0.554480345 | 0.03533255 | -0.08 | -0.16 |
| *Zhx2* | -0.555889391 | 0.0330945 | -0.63 | -0.67 |
| *Elk4* | -0.558368281 | 0.01430232 | -0.54 | -0.54 |
| *St5* | -0.564862522 | 0.00477028 | -0.37 | -1.03 |
| *Tp53i11* | -0.567553195 | 0.02160123 | -0.33 | -0.55 |
| *Creb3l2* | -0.567747136 | 0.03408738 | -0.46 | -0.46 |
| *Dtx3l* | -0.575650837 | 0.03161517 | -0.81 | -1.14 |
| *Ralgds* | -0.580851627 | 0.01232029 | -0.05 | -0.05 |
| *Lrrc61* | -0.583254088 | 0.02966873 | -0.38 | -0.38 |
| *Slc38a2* | -0.58447938 | 0.01728092 | -0.31 | -0.31 |
| *Shmt2* | -0.586234143 | 0.01360338 | -0.74 | -0.74 |
| *App* | -0.587416191 | 0.04094042 | -0.56 | -0.57 |
| *Sesn1* | -0.590581901 | 0.04412474 | -0.55 | -0.55 |
| *Clic4* | -0.597415078 | 0.01501568 | -0.69 | -0.69 |
| *Rps10* | -0.601232707 | 0.01624675 | -0.17 | -0.17 |
| *Furin* | -0.60311562 | 0.00743569 | -0.74 | -0.74 |
| *Ing1* | -0.60322708 | 0.02627635 | -0.51 | -0.64 |
| *Arhgef7* | -0.610356826 | 0.01466965 | -0.24 | -0.24 |
| *Ier5* | -0.613703639 | 0.04704871 | -0.56 | -0.56 |
| *Rassf1* | -0.618740397 | 0.02197663 | -0.22 | -0.22 |
| *Blvrb* | -0.620820189 | 0.03948713 | 0 | -0.41 |
| *RGD1305464* | -0.628034034 | 0.03877064 | -0.6 | -0.6 |
| *Smad6* | -0.631696156 | 0.04810827 | -0.31 | -0.31 |
| *Fam89b* | -0.633415118 | 0.00270271 | -0.2 | -0.34 |
| *Pole3* | -0.633644419 | 0.00760452 | -0.42 | -0.52 |
| *Eif3f* | -0.637259049 | 0.00028496 | -0.14 | -0.14 |
| *Pabpc1* | -0.643642087 | 0.01161343 | -0.22 | -0.22 |
| *Dip2a* | -0.654606396 | 0.00104423 | -0.85 | -0.85 |
| *Lrp4* | -0.654789628 | 0.03235495 | -0.51 | -0.51 |
| *Acvr1b* | -0.662152407 | 0.01149969 | -0.67 | -0.67 |
| *Hist2h2ab* | -0.66261238 | 0.0361666 | -0.66 | -0.66 |
| *Arhgap29* | -0.664580503 | 0.00134351 | -0.24 | -0.24 |
| *Aff1* | -0.664846287 | 0.01892007 | -0.64 | -0.68 |
| *Kitlg* | -0.66595111 | 0.03282955 | -0.45 | -0.45 |
| *Efnb2* | -0.671090842 | 0.03912489 | -1.17 | -2.32 |
| *Sox13* | -0.678258619 | 0.00789719 | -0.2 | -0.8 |
| *C2cd2* | -0.688188839 | 0.0003287 | -0.44 | -0.44 |
| *Zfp496* | -0.692648492 | 0.00336328 | -0.75 | -0.75 |
| *Rai14* | -0.693549463 | 0.01185715 | -0.24 | -0.51 |
| *LOC1003023[illegible]* | -0.693943739 | 0.04001126 | -0.25 | -2.01 |

| | | | | |
|---|---|---|---|---|
| *Cmip* | -0.699038533 | 0.0222173 | -0.07 | -0.39 |
| *Plcg1* | -0.710058787 | 0.00074401 | -0.42 | -0.42 |
| *Myo1c* | -0.717473057 | 0.00046939 | -0.04 | -0.09 |
| *Icam2* | -0.717920157 | 0.03751878 | -1.69 | -1.69 |
| *Amotl2* | -0.731102988 | 0.00185333 | -0.14 | -0.14 |
| *Ybx3* | -0.733637405 | 0.00146725 | -0.2 | -0.2 |
| *H1f0* | -0.734956876 | 0.0059349 | -1.09 | -1.85 |
| *Cdk2* | -0.736460222 | 0.00277606 | -0.7 | -0.7 |
| *Jak1* | -0.741957909 | 0.00100295 | -1.56 | -1.57 |
| *Lurap1l* | -0.743809865 | 0.01832421 | -0.51 | -0.51 |
| *RGD1311739* | -0.758049626 | 0.0153786 | -0.12 | -0.55 |
| *Eif2ak3* | -0.758334499 | 0.01449546 | -0.56 | -0.56 |
| *Cecr2* | -0.759137515 | 0.04351321 | -0.73 | -0.73 |
| *Slc24a3* | -0.767960327 | 0.01375298 | -0.07 | -0.07 |
| *Grpel2* | -0.768971291 | 0.02131018 | -0.66 | -0.66 |
| *Fam65a* | -0.774982935 | 0.00979597 | -0.13 | -0.13 |
| *Cry1* | -0.789138916 | 0.00312912 | -0.54 | -0.54 |
| *RGD1309079* | -0.789932723 | 0.00156081 | -0.61 | -0.61 |
| *Plxna2* | -0.791823803 | 0.00122309 | -0.48 | -0.48 |
| *Nuak1* | -0.79352834 | 0.01421144 | -0.18 | 1.21 |
| *Tnrc18* | -0.794221416 | 0.0262901 | -0.6 | -0.6 |
| *Epha7* | -0.798921585 | 0.03522436 | -0.43 | -0.48 |
| *Klf10* | -0.805622276 | 0.00941918 | -0.2 | 0.79 |
| *Magi3* | -0.807743329 | 0.00072898 | -0.17 | -0.17 |
| *Mdfic* | -0.809150393 | 0.00084605 | -0.77 | -0.77 |
| *Irs1* | -0.81150711 | 0.00320098 | -0.25 | -0.25 |
| *Dxo* | -0.820578057 | 0.01049146 | -0.4 | -0.4 |
| *Bmp1* | -0.82286178 | 6.71E-05 | -0.28 | -0.28 |
| *Ak3* | -0.826042673 | 0.00130352 | -0.77 | -0.77 |
| *Slc7a1* | -0.83012263 | 0.00559119 | -0.57 | -0.58 |
| *Mast3* | -0.83018293 | 0.00452542 | -0.34 | -0.46 |
| *Crot* | -0.830866518 | 0.00202087 | -1.35 | -2.64 |
| *Ccdc102a* | -0.841636308 | 0.02780011 | -1 | -1 |
| *Camta1* | -0.843346321 | 0.00584013 | -0.52 | -0.52 |
| *Pdk1* | -0.845212132 | 0.0046138 | -0.54 | -0.54 |
| *Galnt10* | -0.848730502 | 0.00368188 | -0.71 | -0.86 |
| *Dtnb* | -0.849271245 | 0.00344356 | -0.39 | -0.39 |
| *Nudt4* | -0.85363613 | 0.00015237 | -0.73 | -0.73 |
| *Sypl1* | -0.855449655 | 5.19E-05 | -0.46 | -0.46 |
| *Spry2* | -0.85974981 | 0.00293138 | -0.64 | -0.64 |
| *Gtf3c5* | -0.860265788 | 0.00980157 | -0.09 | -0.67 |
| *Msrb1* | -0.860667403 | 0.00095539 | -0.06 | -0.21 |
| *Sav1* | -0.862429088 | 2.98E-05 | -0.18 | -0.92 |
| *Plp2* | -0.870762519 | 2.22E-05 | -0.03 | -0.62 |
| *Ly6e* | -0.872498434 | 3.67E-06 | -0.66 | -0.66 |

| | | | | |
|---|---|---|---|---|
| *S100a10* | -0.875111148 | 0.00124078 | -0.66 | -3.28 |
| *Swap70* | -0.877489728 | 0.0304867 | -1.09 | -1.09 |
| *Adap2* | -0.878313977 | 0.02183961 | -0.62 | -0.62 |
| *Tmco4* | -0.882050499 | 0.01078263 | -0.26 | -0.67 |
| *Ncor2* | -0.883536242 | 0.03912598 | -0.22 | -0.69 |
| *Csrp1* | -0.885257501 | 0.00044026 | -0.32 | -0.32 |
| *Lbh* | -0.887932801 | 0.02226293 | -0.5 | -0.5 |
| *Agpat2* | -0.894679995 | 0.00020762 | -0.48 | -0.54 |
| *Wdtc1* | -0.900903994 | 0.00125535 | -0.65 | -0.65 |
| *Rcn1* | -0.902605655 | 0.0001281 | -0.57 | -0.58 |
| *Sufu* | -0.913265734 | 0.04351321 | -0.31 | -0.31 |
| *Frmd6* | -0.915014419 | 0.00027145 | -0.36 | -0.36 |
| *Foxa3* | -0.918145584 | 0.02373804 | -0.25 | -0.87 |
| *Id2* | -0.93324176 | 1.33E-05 | -0.08 | -0.63 |
| *Klf9* | -0.938070907 | 4.49E-05 | -0.38 | -0.4 |
| *Wdr62* | -0.956089113 | 0.0195313 | -0.15 | -0.37 |
| *Sh3d21* | -0.962017505 | 0.02945868 | -0.52 | -0.52 |
| *Slc12a2* | -0.964211848 | 0.00062592 | -0.92 | -0.92 |
| *Sfxn2* | -0.968582527 | 0.03910276 | -0.48 | -0.53 |
| *Bhlhe40* | -0.971051041 | 0.00072489 | -0.28 | -0.28 |
| *Pim1* | -0.971266213 | 0.00773284 | -0.15 | -0.15 |
| *Rab43* | -0.973748019 | 0.00116502 | -0.62 | -0.62 |
| *Echdc3* | -0.976513986 | 0.04565245 | -0.57 | -0.58 |
| *Htr7* | -0.977034799 | 0.03253518 | -1.44 | -1.44 |
| *Il4r* | -0.984827237 | 0.01092893 | -1.18 | -1.18 |
| *Prr7* | -0.987815689 | 0.04596642 | -0.16 | -0.16 |
| *Fam213a* | -0.992449011 | 0.00369749 | -0.46 | -0.46 |
| *Stap2* | -0.996934247 | 0.01214516 | -0.11 | -0.11 |
| *Rps27l* | -1.003090709 | 0.00344574 | -0.45 | -0.45 |
| *Pla2g4a* | -1.028915713 | 0.00037084 | -0.51 | -0.51 |
| *Bckdk* | -1.033536092 | 0.00303558 | -0.26 | -0.26 |
| *Cited2* | -1.039846891 | 5.03E-07 | -0.16 | -0.16 |
| *Il7* | -1.041226552 | 0.02619054 | -0.06 | -0.06 |
| *Parva* | -1.041276275 | 1.13E-07 | -0.62 | -0.62 |
| *Pik3c2b* | -1.052713822 | 0.00029704 | -0.52 | -0.52 |
| *Man2a1* | -1.055219651 | 1.40E-06 | -0.72 | -0.72 |
| *Ifi47* | -1.058229532 | 0.02460999 | -1.11 | -1.11 |
| *Bcl9l* | -1.062374869 | 0.00382632 | -0.5 | -0.55 |
| *Nipsnap3b* | -1.065063399 | 0.00186488 | -0.88 | -0.94 |
| *Bckdhb* | -1.067873591 | 0.00959061 | -0.43 | -0.43 |
| *RT1-T24-3* | -1.068434047 | 0.00212809 | -0.75 | -0.75 |
| *Ppp1r16b* | -1.074655475 | 0.04471178 | -0.54 | -0.54 |
| *Hlx* | -1.079572021 | 0.00123092 | -0.82 | -0.82 |
| *Nt5dc2* | -1.07978099 | 0.00397321 | -0.15 | -0.16 |
| *Tppp* | -1.082893736 | 0.0140815 | -0.06 | -0.31 |

| | | | | |
|---|---|---|---|---|
| *Itga6* | -1.085755214 | 3.78E-09 | -0.48 | -0.48 |
| *Hes1* | -1.087010058 | 4.51E-06 | -0.21 | -0.41 |
| *Myrf* | -1.087246896 | 0.00171001 | -0.7 | -0.7 |
| *Kank3* | -1.088122559 | 0.00026376 | -0.85 | -0.85 |
| *Foxo3* | -1.090617461 | 7.32E-08 | -2.21 | -2.21 |
| *Fam46c* | -1.09204085 | 0.0092868 | -0.67 | -0.67 |
| *Grasp* | -1.107953631 | 0.00055874 | -0.28 | -0.29 |
| *Tmem47* | -1.109680317 | 3.52E-10 | -0.69 | -0.69 |
| *Fhl3* | -1.115540375 | 0.03324952 | -0.34 | -0.34 |
| *Rab13* | -1.11603662 | 7.41E-06 | -0.56 | -0.57 |
| *Bach1* | -1.118184694 | 6.75E-07 | -0.52 | -0.52 |
| *Ier2* | -1.130954746 | 9.15E-06 | -0.17 | -0.17 |
| *Rhbdf2* | -1.133227877 | 0.00038864 | -0.19 | -0.8 |
| *Fli1* | -1.140736062 | 0.00364997 | -0.74 | -0.74 |
| *Emp3* | -1.143100941 | 0.00045625 | -0.25 | -0.25 |
| *Spry1* | -1.146970554 | 1.08E-08 | -0.48 | -0.57 |
| *Tgfb1* | -1.159651486 | 0.00896302 | -0.5 | -0.5 |
| *Hist1h1a* | -1.162012502 | 0.01807037 | -0.16 | -0.61 |
| *Sema3c* | -1.174572299 | 1.44E-06 | -0.39 | -0.39 |
| *Pycr1* | -1.175553896 | 0.02054619 | -1.3 | -1.3 |
| *Eda* | -1.180179305 | 0.00295874 | -0.24 | -0.24 |
| *Fzd4* | -1.184431959 | 0.00026032 | -0.08 | -0.08 |
| *Myadm* | -1.194350479 | 5.78E-06 | -0.7 | -0.7 |
| *Nfkbia* | -1.200498437 | 0.00047424 | -0.5 | -0.5 |
| *Maff* | -1.20407648 | 0.00127983 | -0.52 | -0.52 |
| *Sertad1* | -1.208787215 | 0.00075643 | -0.7 | -0.7 |
| *Slc28a2* | -1.217725326 | 0.00403673 | -1.05 | -1.05 |
| *Acer2* | -1.22142507 | 0.00010723 | -0.59 | -0.59 |
| *Bmf* | -1.224441123 | 0.00020087 | -0.65 | -0.65 |
| *Raver2* | -1.228053523 | 0.0188466 | -0.59 | -0.59 |
| *Jun* | -1.229126539 | 4.76E-06 | -0.44 | -0.44 |
| *Usp2* | -1.236424208 | 0.01003703 | -0.15 | -0.97 |
| *Syngr2* | -1.237354708 | 1.60E-09 | -0.57 | -0.58 |
| *Map3k8* | -1.239512569 | 0.00118494 | -0.6 | -0.6 |
| *Rasip1* | -1.24196551 | 3.71E-06 | -0.46 | -0.46 |
| *Mpst* | -1.244916433 | 2.20E-06 | -0.24 | -0.24 |
| *Dmd* | -1.246818236 | 2.23E-08 | -0.17 | -0.17 |
| *Plekhg2* | -1.24702968 | 0.00292968 | -0.9 | -0.9 |
| *Peli2* | -1.247294075 | 3.51E-06 | -0.67 | -0.67 |
| *Dnase2* | -1.251288788 | 6.08E-07 | -0.47 | -0.47 |
| *Lrrc8c* | -1.252752743 | 0.00565922 | -0.53 | -0.53 |
| *B4galnt1* | -1.259515055 | 0.00471749 | -0.19 | -1.35 |
| *Tgfb3* | -1.265537223 | 3.28E-05 | -0.66 | -0.67 |
| *Card10* | -1.274611687 | 2.73E-10 | -0.22 | -0.22 |
| *Zfp423* | -1.282884942 | 0.001365 | -0.69 | -0.69 |

| | | | | |
|---|---|---|---|---|
| *Arhgap31* | -1.283902812 | 9.60E-05 | -0.28 | -0.28 |
| *Anxa1* | -1.294944627 | 9.93E-05 | -2.22 | -2.22 |
| *Atp7b* | -1.295969911 | 0.001365 | -0.15 | -0.15 |
| *Sik1* | -1.309034007 | 2.01E-06 | -0.18 | -0.18 |
| *Ptprv* | -1.311692073 | 0.00920613 | -1.8 | -1.8 |
| *Zfp503* | -1.313976995 | 7.76E-08 | -0.26 | -0.26 |
| *Cebpb* | -1.315331664 | 8.23E-05 | -0.22 | -0.22 |
| *Enpp1* | -1.32611036 | 7.86E-11 | -0.66 | -0.66 |
| *Gucd1* | -1.34322789 | 8.13E-06 | -0.73 | -0.73 |
| *Pter* | -1.352861001 | 0.00051051 | -0.82 | -0.82 |
| *Cp* | -1.355824123 | 0.00034139 | -1.04 | -1.04 |
| *Vcam1* | -1.3646969 | 0.00581823 | -2.06 | -2.07 |
| *Rnpepl1* | -1.366252669 | 9.37E-07 | -0.3 | -0.3 |
| *Acsf2* | -1.378099947 | 2.28E-05 | -0.56 | -0.57 |
| *Klf4* | -1.388647186 | 0.00029146 | -0.26 | -0.26 |
| *Prcp* | -1.390322546 | 6.02E-10 | -0.69 | -0.69 |
| *Kazald1* | -1.396654443 | 9.57E-05 | -1.17 | -1.17 |
| *Rab38* | -1.407731761 | 0.00743569 | -0.34 | -1.13 |
| *Ifrd1* | -1.418614869 | 5.51E-10 | -0.74 | -1.05 |
| *Pla2g16* | -1.437830246 | 1.38E-05 | -0.52 | -0.52 |
| *Cgnl1* | -1.438060855 | 1.24E-07 | -0.54 | -0.54 |
| *Ebf1* | -1.463904911 | 8.85E-06 | -0.57 | -0.58 |
| *Mocos* | -1.466863936 | 0.00886711 | -0.19 | -0.19 |
| *Maf* | -1.471174313 | 0.00161237 | -0.89 | -1.64 |
| *Slc16a1* | -1.484798565 | 2.44E-08 | -0.5 | -0.5 |
| *Kdm6b* | -1.489378962 | 0.00126341 | 0.19 | -0.67 |
| *Madcam1* | -1.493166081 | 0.0005662 | -0.57 | -0.58 |
| *Zcchc24* | -1.510666279 | 3.44E-08 | -0.61 | -0.7 |
| *Vipr1* | -1.526789128 | 0.0055554 | -0.62 | -0.62 |
| *Nfib* | -1.532297934 | 1.07E-09 | -0.73 | -0.73 |
| *Gcat* | -1.552489975 | 0.00057096 | -1.09 | -1.85 |
| *Piezo1* | -1.586419475 | 8.14E-07 | -0.34 | -0.34 |
| *Msra* | -1.599664596 | 0.00061984 | -0.52 | -0.52 |
| *Gli1* | -1.606624202 | 0.03261855 | -0.15 | -0.72 |
| *Mospd1* | -1.624294049 | 1.91E-06 | -1.39 | -1.39 |
| *Prkcg* | -1.631194285 | 0.0295991 | -0.56 | -0.57 |
| *Jam2* | -1.66859177 | 2.36E-08 | -0.14 | -0.14 |
| *Nr2f2* | -1.671010212 | 1.85E-07 | -0.08 | -0.34 |
| *Zfp36l1* | -1.688270945 | 3.21E-09 | 0 | -0.93 |
| *Aldh1a2* | -1.700963545 | 2.91E-06 | -0.47 | -0.47 |
| *Fam210b* | -1.704860107 | 0.00024337 | -1.33 | -1.33 |
| *Igsf3* | -1.708025164 | 9.00E-09 | -0.11 | -0.11 |
| *Ggh* | -1.710103259 | 0.00253942 | -0.63 | -0.63 |
| *Galnt6* | -1.710440364 | 0.01313033 | -2.19 | -2.19 |
| *ST7* | -1.715247045 | 1.07E-06 | -0.55 | -0.55 |

| | | | | |
|---|---|---|---|---|
| *Meox2* | -1.736574459 | 2.40E-07 | -1.77 | -1.77 |
| *Ptgfrn* | -1.741551136 | 1.33E-18 | -0.63 | -0.63 |
| *Cyp1b1* | -1.741808932 | 0.04872477 | -0.26 | -0.61 |
| *Socs3* | -1.765289037 | 3.98E-05 | -0.9 | -0.9 |
| *Trib3* | -1.766101276 | 2.13E-05 | -0.61 | -0.61 |
| *Tcf15* | -1.793043006 | 0.00123452 | -0.62 | -0.62 |
| *Zfp521* | -1.813243899 | 3.18E-09 | -0.56 | -0.56 |
| *Duox1* | -1.835882635 | 0.00558628 | -1.36 | -1.36 |
| *Csrnp1* | -1.843362632 | 4.63E-07 | 0.11 | -1.29 |
| *Tnfsf9* | -1.844786318 | 0.02105566 | -1.89 | -1.89 |
| *Parp16* | -1.861930019 | 1.12E-06 | -0.71 | -0.71 |
| *Chmp4c* | -1.904866367 | 5.73E-09 | -0.77 | -0.77 |
| *Duoxa1* | -1.905516136 | 0.00152481 | -1.36 | -1.36 |
| *Ptgs2* | -1.911953079 | 1.42E-05 | -0.29 | -1.58 |
| *Ctf1* | -1.913105365 | 0.00012708 | -0.59 | -0.59 |
| *Ppard* | -1.971175032 | 7.32E-11 | -0.48 | -0.48 |
| *Rnd1* | -1.99217072 | 9.09E-05 | 0.02 | -0.63 |
| *Atf3* | -2.00187558 | 1.73E-06 | -0.74 | -0.74 |
| *Pfkfb3* | -2.003393613 | 1.17E-05 | -0.12 | -0.31 |
| *Col3a1* | -2.019362187 | 6.61E-06 | -1.51 | -1.67 |
| *Flrt2* | -2.072838638 | 3.77E-10 | 0 | -0.14 |
| *Scnn1g* | -2.089601282 | 0.00254228 | -0.43 | -0.43 |
| *Tnf* | -2.101881623 | 0.00201979 | -1.47 | -1.62 |
| *Ltc4s* | -2.114633924 | 0.0065235 | 0.13 | -0.58 |
| *Cebpa* | -2.129374491 | 4.82E-11 | -0.73 | -0.73 |
| *Slc1a3* | -2.135732156 | 3.60E-12 | -0.56 | -0.56 |
| *Pabpc4* | -2.142179844 | 2.86E-10 | -0.7 | -0.7 |
| *Egr2* | -2.197794611 | 4.09E-06 | -0.64 | -0.64 |
| *Slc6a9* | -2.21571648 | 2.79E-11 | -0.38 | -0.38 |
| *Nr4a3* | -2.297921651 | 0.00016048 | -0.5 | -0.68 |
| *Bmp2* | -2.31546098 | 0.00014612 | -0.89 | -0.89 |
| *Slc25a45* | -2.439941589 | 2.40E-08 | 0 | -0.04 |
| *Mpz* | -2.493595543 | 0.00267129 | -0.55 | -0.55 |
| *Qsox1* | -2.498825371 | 4.14E-10 | -0.37 | -0.37 |
| *Chrna5* | -2.550006127 | 0.01320737 | -0.28 | -0.28 |
| *Rnase4* | -2.557966136 | 2.25E-14 | -0.57 | -0.97 |
| *Gfi1* | -2.559409137 | 5.21E-07 | -0.82 | -0.82 |
| *Fam46b* | -2.572405007 | 8.62E-06 | -0.27 | -0.27 |
| *Cish* | -2.649561686 | 1.32E-17 | -0.71 | -0.71 |
| *Fst* | -2.838674625 | 0.0018088 | -0.82 | -0.82 |
| *Tac4* | -2.846972199 | 9.95E-05 | -0.78 | -0.78 |
| *Grpr* | -3.006503666 | 5.61E-06 | -1.48 | -1.48 |
| *Mrap2* | -3.076840657 | 1.11E-07 | -0.8 | -0.8 |
| *Depdc7* | -3.107801768 | 1.04E-11 | -0.6 | -0.6 |
| *Tst* | -3.126253909 | 9.28E-14 | -0.24 | -0.24 |

| | | | | |
|---|---|---|---|---|
| *Icam4* | -3.129070676 | 0.00398866 | 0.02 | -1.2 |
| *Ddah1* | -3.395549229 | 1.07E-11 | -0.38 | -0.38 |
| *Gls2* | -3.500050728 | 1.07E-11 | -0.48 | -2.29 |
| *Gpc5* | -3.617546985 | 0.01371774 | -0.94 | -0.94 |
| *Rtp3* | -4.323949784 | 7.49E-08 | -0.94 | -0.94 |
| *Cpa2* | -5.647404409 | 3.28E-11 | -1.75 | -1.75 |
| *Serpini2* | -5.72173296 | 6.49E-15 | -1.7 | -1.7 |
| *Cel* | -5.937671128 | 4.58E-11 | -0.83 | -0.83 |
| *Amy2a3* | -6.053673268 | 6.27E-09 | -2.06 | -2.06 |
| *Amy1a* | -7.260514885 | 1.32E-17 | -2.19 | -2.19 |
| *Clpsl2* | -8.31234808 | 2.22E-09 | -0.28 | -0.28 |

**hanges**

**Supplemental Table S4 Differentially Expressed Genes with Consistent H3K27me3 Histone Mark Ch**

**2-wk Islets**

| **Gene** | **RNAseq_logFC** | **RNAseq_FDR** | **H3K27me3_avgpeak_logFC** | **H3K27me3_maxpeak_logFC** |
|---|---|---|---|---|
| *Ncan* | 3.648915424 | 0.00663879 | -0.99 | -0.99 |
| *Elavl3* | 2.223356899 | 0.0303281 | -0.65 | -0.65 |
| *Dok5* | 1.667380688 | 0.04358085 | -0.75 | -0.75 |
| *Nefl* | 1.632359682 | 0.00972503 | -0.62 | -0.62 |
| *Cxcl2* | 1.54079926 | 0.02603199 | -0.85 | -0.85 |
| *Lrrc10b* | 1.18760652 | 0.01009017 | -0.75 | -0.75 |
| *Gap43* | 1.167820841 | 0.03204959 | -1.12 | -1.12 |
| *Tubb3* | 1.096496002 | 0.00036042 | -0.92 | -0.92 |
| *Acta1* | 1.076819231 | 0.02190215 | -0.72 | -0.72 |
| *Dpysl3* | 1.035289653 | 0.01333355 | -0.82 | -0.82 |
| *Dgkg* | 1.035101469 | 0.03141833 | -1.34 | -1.34 |
| *LOC691995* | 0.999573675 | 0.01959395 | -0.86 | -0.86 |
| *Itgb3* | 0.929352943 | 0.0414565 | -1 | -1.24 |
| *Tubb2b* | 0.90326132 | 0.00273421 | -0.81 | -0.81 |
| *Vim* | 0.868352027 | 0.03971334 | -0.76 | -0.76 |
| *Tifa* | 0.801691896 | 0.04079356 | -0.72 | -0.77 |
| *Mark1* | 0.789310033 | 0.00916491 | -0.78 | -0.78 |
| *Reep1* | 0.780811539 | 0.03086611 | -1.02 | -1.02 |
| *Rgs4* | 0.770213566 | 0.00233662 | -0.85 | -0.91 |
| *Adra2a* | 0.719802272 | 0.04690859 | -0.89 | -0.89 |
| *Flnc* | 0.706764775 | 0.02895713 | -0.72 | -0.8 |
| *Trib2* | 0.685050163 | 0.0424242 | -0.96 | -0.96 |
| *Ednra* | 0.662812397 | 0.04114461 | -1.11 | -1.11 |
| *Tubgcp6* | -0.463612678 | 0.02539191 | 0.92 | 0.92 |
| *Gramd1a* | -0.55015476 | 0.00653472 | 1.5 | 1.5 |
| *Igsf11* | -0.584956315 | 0.02638313 | 1.07 | 1.07 |
| *Smad3* | -0.619422655 | 0.04008342 | 0.89 | 0.89 |
| *Myo5b* | -0.64606543 | 0.03207989 | 0.94 | 0.94 |
| *Pck2* | -0.688530259 | 0.02099762 | 1.52 | 1.52 |
| *Synj2* | -0.737416273 | 0.02545271 | 1.54 | 1.54 |
| *Hdac10* | -0.761218721 | 0.00237374 | 0.92 | 0.92 |
| *Litaf* | -0.774803464 | 0.02186184 | 1.1 | 1.1 |
| *Dap* | -0.790474972 | 0.0407863 | 1.03 | 1.03 |
| *Clmn* | -0.806639705 | 0.04391289 | 1.15 | 1.16 |
| *Tfap4* | -0.809996487 | 0.01300388 | 1.35 | 1.35 |
| *Fuca2* | -0.8165312 | 0.02099762 | 0.94 | 0.94 |
| *Fam110a* | -0.835913618 | 0.02156974 | 1.35 | 1.35 |
| *Eif2ak3* | -0.848004943 | 0.0136611 | 1.34 | 1.34 |
| *Rcbtb1* | -0.857643991 | 0.00653472 | 1.11 | 1.11 |
| *Abcc10* | -0.862413453 | 0.01009017 | 1.23 | 1.23 |
| *Hm13* | -0.889206654 | 0.01844836 | 0.92 | 0.92 |

| | | | | |
|---|---|---|---|---|
| *Pde4c* | -0.89434459 | 0.03414726 | 1.06 | 1.06 |
| *Tjp3* | -0.896715731 | 0.00099345 | 1.14 | 1.14 |
| *Hipk2* | -0.946474136 | 0.00119744 | 1.11 | 1.11 |
| *Acpp* | -0.9883218 | 0.00912202 | 1.32 | 1.32 |
| *Parp16* | -1.012857751 | 0.03559849 | 0.89 | 0.89 |
| *Gstt3* | -1.026732465 | 0.03532694 | 0.98 | 0.98 |
| *Camsap3* | -1.065886649 | 0.00229421 | 1.3 | 1.3 |
| *Acadsb* | -1.130369335 | 0.0004992 | 1.03 | 1.03 |
| *Cd24* | -1.137541004 | 0.00626481 | 0.88 | 0.88 |
| *Pecr* | -1.202931678 | 0.01059422 | 0.91 | 0.91 |
| *Macrod1* | -1.292606444 | 0.00056658 | 0.96 | 0.96 |
| *Cbfa2t3* | -1.313308786 | 0.00233662 | 0.89 | 0.89 |
| *Kirrel2* | -1.430126527 | 0.02384424 | 1 | 1 |
| *Fam83f* | -1.45962118 | 3.69E-05 | 0.99 | 0.99 |
| *Myrf* | -1.683371489 | 2.57E-06 | 1.22 | 1.22 |
| *LOC257642* | -1.909379897 | 0.02634348 | 1.03 | 1.11 |
| *Rab26* | -2.2713485 | 9.27E-05 | 0.97 | 0.97 |
| *Klk9* | -2.307865355 | 0.0214732 | 0.94 | 0.94 |
| *Cela1* | -3.015740715 | 0.00229421 | 1.17 | 1.17 |
| *Cpa4* | -3.555341045 | 0.03202966 | 1 | 1 |

**10-wk Islets**

| Gene | RNAseq_logFC | RNAseq_FDR | H3K27me3_avgpeak_logFC | H3K27me3_maxpeak_logFC |
|---|---|---|---|---|
| *Lhfpl5* | 2.002878365 | 1.51E-07 | -1.01 | -1.26 |
| *Ssmem1* | 1.490087662 | 0.00863035 | -1.39 | -1.39 |
| *Clcnkb* | 1.155699503 | 0.04658238 | -0.64 | -0.68 |
| *Kcnk2* | 1.126994395 | 0.0218037 | -0.84 | -1.85 |
| *Asic1* | 0.841823893 | 0.04300371 | -0.61 | -0.67 |
| *Trpm5* | 0.830189818 | 0.02145042 | -0.78 | -0.88 |
| *Nova1* | 0.811799613 | 0.03681303 | -1.26 | -1.26 |
| *Abat* | 0.752327289 | 0.00603307 | -0.76 | -1.74 |
| *Zfp709* | 0.723479523 | 0.00185907 | -1.32 | -1.32 |
| *Pcdh9* | 0.685965596 | 0.00239237 | -1.14 | -1.14 |
| *Ntan1* | 0.679783858 | 0.00058467 | -1.67 | -1.67 |
| *Scoc* | 0.671834071 | 0.00029213 | -0.99 | -0.99 |
| *Scn2b* | 0.647775867 | 0.04260911 | -1.15 | -1.16 |
| *Elovl6* | 0.628365777 | 0.015563 | -1.96 | -1.96 |
| *Akirin1* | 0.612719127 | 0.00328178 | -0.85 | -0.85 |
| *Foxa2* | 0.53144823 | 0.01209825 | -0.72 | -0.72 |
| *Gorab* | 0.529991231 | 0.02924269 | -1.59 | -1.59 |
| *Tfam* | 0.501433582 | 0.01479307 | -0.95 | -0.95 |
| *Ube2e3* | 0.472789111 | 0.01103024 | -1.15 | -1.16 |
| *Eif4e* | 0.472758142 | 0.01488435 | -1.34 | -1.34 |
| *Ncoa5* | 0.472010357 | 0.01853628 | -0.85 | -0.85 |
| *Mcf2l* | 0.466933434 | 0.05002442 | -1.06 | -1.16 |

| | | | | |
|---|---|---|---|---|
| *Crbn* | 0.453010061 | 0.04094042 | -0.86 | -0.86 |
| *Fbxo3* | 0.430137638 | 0.04003699 | -0.71 | -0.71 |
| *Cald1* | -0.509877185 | 0.04902462 | 1.67 | 1.67 |
| *Spns2* | -0.540136515 | 0.03385409 | 1.28 | 1.33 |
| *Fbn2* | -0.552084272 | 0.04290295 | 0.47 | 1.24 |
| *Tp53i11* | -0.567553195 | 0.02160123 | 2.14 | 2.14 |
| *Ajuba* | -0.645733927 | 0.01788195 | 1.12 | 1.13 |
| *Pdgfrb* | -0.65671955 | 0.0461962 | 0 | 1.11 |
| *Nrp2* | -0.664366091 | 0.04003378 | 1.35 | 1.43 |
| *Gja1* | -0.667048503 | 0.0305174 | 1.26 | 1.26 |
| *Vstm4* | -0.682041967 | 0.03520103 | 1.34 | 1.34 |
| *Slc9a3r2* | -0.708321586 | 0.00428498 | 2.25 | 2.25 |
| *Ednra* | -0.711539238 | 0.01203891 | 1.39 | 1.39 |
| *Sema3f* | -0.728689143 | 0.0031361 | 0.88 | 0.88 |
| *Clec14a* | -0.728867157 | 0.00863035 | 1.18 | 1.18 |
| *Sparc* | -0.735899187 | 0.0492313 | 1.12 | 1.13 |
| *Tnfrsf1b* | -0.7486814 | 0.03981488 | 0.91 | 1 |
| *Bmp6* | -0.763322263 | 0.01694953 | 0.71 | 1.19 |
| *Egflam* | -0.766936348 | 0.00755144 | 1.43 | 1.43 |
| *Rftn1* | -0.777000512 | 0.03485029 | 1.22 | 1.22 |
| *Sox18* | -0.783799197 | 0.02321664 | 0.41 | 1.16 |
| *Sulf2* | -0.793622803 | 0.00145632 | 0.6 | 1.47 |
| *Trip6* | -0.794823739 | 0.00077336 | 1.49 | 1.49 |
| *Susd4* | -0.80540051 | 0.0174643 | 0.87 | 2.64 |
| *Palm* | -0.8058734 | 0.00493522 | 1.51 | 1.51 |
| *Frzb* | -0.818751023 | 0.03533255 | 1 | 1 |
| *Bmp1* | -0.82286178 | 6.71E-05 | 1.26 | 1.26 |
| *Ltbp1* | -0.839372612 | 0.0168117 | 0.29 | 1.27 |
| *Ccdc102a* | -0.841636308 | 0.02780011 | 1.01 | 1.08 |
| *Cmtm3* | -0.844401989 | 0.03530707 | 1.09 | 1.09 |
| *Egfl7* | -0.845096816 | 0.0021872 | 1.25 | 1.25 |
| *Nes* | -0.847261239 | 0.00063044 | 1.62 | 1.62 |
| *Prkch* | -0.849186209 | 0.01560548 | 1.06 | 1.06 |
| *Prkg1* | -0.859784681 | 0.01448208 | 1.31 | 1.31 |
| *Gstm5* | -0.862877471 | 0.02659402 | 2.53 | 2.53 |
| *Des* | -0.868699611 | 0.00428892 | -0.05 | 1.45 |
| *Cpxm2* | -0.880082483 | 0.03462187 | 1.03 | 1.05 |
| *Syde1* | -0.886897081 | 0.00571808 | 2.14 | 2.68 |
| *Rcn1* | -0.902605655 | 0.0001281 | 1.88 | 1.88 |
| *Pltp* | -0.905296964 | 0.00122309 | 1.56 | 1.57 |
| *Ppp1r18* | -0.912740895 | 0.03946105 | 1.34 | 1.34 |
| *Tpm2* | -0.933641621 | 0.03007938 | 2.86 | 2.86 |
| *Sphk1* | -0.939861492 | 0.02503378 | -0.21 | 1.03 |
| *Akap2* | -0.942555925 | 0.0022634 | 1.81 | 1.81 |
| *Rnf144a* | -0.942599462 | 0.01739497 | 1.52 | 1.52 |

| | | | | |
|---|---|---|---|---|
| *Smoc2* | -0.942959777 | 0.00460628 | -0.04 | 1.12 |
| *Slco2b1* | -0.943973578 | 0.01842786 | 1.35 | 1.41 |
| *Cxcl12* | -0.948393537 | 0.00617361 | 1.11 | 1.21 |
| *Emp2* | -0.979178246 | 3.10E-05 | 1.37 | 1.37 |
| *Adamts7* | -0.980494637 | 0.01147633 | 1.08 | 1.08 |
| *RGD1561157* | -0.982434742 | 0.04035082 | 1.04 | 1.04 |
| *Prr7* | -0.987815689 | 0.04596642 | 1.46 | 1.89 |
| *Erbb2* | -0.990710107 | 0.00748063 | 0.2 | 1.25 |
| *Neurl1b* | -0.994829047 | 0.00309471 | 1.15 | 1.29 |
| *Etnk2* | -0.996281406 | 0.04869006 | 1.07 | 1.07 |
| *Hspa12b* | -0.996650401 | 0.00061247 | 1.55 | 1.55 |
| *Ppic* | -1.003046684 | 0.00021442 | 1.04 | 1.04 |
| *Trim47* | -1.009348622 | 0.00353603 | 0.75 | 0.75 |
| *Ptprm* | -1.016718362 | 3.15E-06 | 1.56 | 1.57 |
| *Gja5* | -1.019839005 | 0.0151493 | 2.19 | 2.19 |
| *Pdlim2* | -1.023645062 | 0.02678702 | -0.03 | 0.81 |
| *Acvrl1* | -1.027401971 | 0.00176993 | 1.85 | 1.85 |
| *Slc2a3* | -1.029261549 | 0.01487781 | 0.98 | 1.02 |
| *Pcsk5* | -1.032889474 | 0.00017878 | 1.18 | 1.18 |
| *Apold1* | -1.033039435 | 0.03209546 | 1.8 | 1.8 |
| *Nkx2-3* | -1.035324677 | 0.01558059 | 1.21 | 1.22 |
| *Efs* | -1.038438287 | 0.01881604 | 1.21 | 1.21 |
| *Ace3* | -1.044844129 | 0.0024217 | 1.22 | 1.22 |
| *Rasl11a* | -1.045837446 | 0.0248708 | 1.48 | 1.48 |
| *Loxl1* | -1.050940321 | 1.13E-05 | 1.52 | 2.01 |
| *Nos3* | -1.056343551 | 0.0042321 | 1.24 | 1.24 |
| *Pcsk6* | -1.05703363 | 7.05E-06 | 1.3 | 1.3 |
| *Hic1* | -1.057102026 | 0.00215421 | 0.97 | 0.97 |
| *Slfn2* | -1.058519037 | 0.04003378 | 1.56 | 1.57 |
| *Ppp1r16b* | -1.074655475 | 0.04471178 | 1.22 | 1.22 |
| *Fat4* | -1.078345763 | 9.40E-05 | 0.32 | 1.22 |
| *Cmklr1* | -1.078972042 | 0.01042168 | -0.06 | 1.18 |
| *C1qtnf1* | -1.079144154 | 5.31E-05 | 1.65 | 1.65 |
| *Hlx* | -1.079572021 | 0.00123092 | 1.22 | 1.5 |
| *Slco2a1* | -1.080889079 | 0.01041705 | 1.33 | 1.33 |
| *Gadd45g* | -1.081576802 | 3.61E-06 | 1.44 | 1.44 |
| *Pth1r* | -1.084063988 | 0.00694352 | 0.06 | 1.34 |
| *Kank3* | -1.088122559 | 0.00026376 | 1.35 | 1.35 |
| *Cpz* | -1.095457587 | 0.00616031 | 0.12 | 1.29 |
| *Rasl11b* | -1.095516851 | 0.00177429 | 0.25 | 1.41 |
| *Mmp17* | -1.101845899 | 0.00039425 | 1.23 | 1.23 |
| *Apol3* | -1.104024552 | 0.00968211 | 0.94 | 0.94 |
| *Notch4* | -1.109287106 | 0.00056812 | 1.12 | 1.13 |
| *Spon2* | -1.110067718 | 0.00460002 | 2.02 | 2.03 |
| *Evc2* | -1.114717977 | 0.00344356 | 1.71 | 1.71 |

| | | | | |
|---|---|---|---|---|
| *Plvap* | -1.117958781 | 8.84E-05 | 1.91 | 1.91 |
| *Col15a1* | -1.11935791 | 2.47E-05 | 1.08 | 1.53 |
| *Jph2* | -1.123177884 | 0.01452999 | 1.35 | 1.35 |
| *Cplx1* | -1.125321828 | 0.04094042 | 1.25 | 1.25 |
| *Pde2a* | -1.130145734 | 0.00048014 | 0.03 | 1.46 |
| *Fli1* | -1.140736062 | 0.00364997 | 0.44 | 1.19 |
| *Tubb6* | -1.145735089 | 0.00086419 | 1.29 | 1.29 |
| *Gpx2* | -1.150630846 | 0.00917037 | 1.29 | 1.49 |
| *Clec2l* | -1.152224514 | 0.01531735 | -0.35 | 1.08 |
| *Mrc2* | -1.154853211 | 0.00032768 | 1.11 | 1.11 |
| *Epha2* | -1.158144425 | 0.00010946 | 0.31 | 1.37 |
| *Tgfb1* | -1.159651486 | 0.00896302 | 0.65 | 1.27 |
| *Cdh6* | -1.161139736 | 2.02E-05 | 0.37 | 1.4 |
| *Grip2* | -1.165409923 | 0.01810474 | 1.36 | 1.44 |
| *Adcy4* | -1.16777101 | 0.00201998 | 1.14 | 1.27 |
| *Paqr4* | -1.168465645 | 0.00025382 | 1.26 | 1.49 |
| *Notch1* | -1.173601323 | 0.00037839 | 1.71 | 1.86 |
| *Plxdc2* | -1.177692173 | 1.09E-06 | 1.46 | 1.46 |
| *Krt80* | -1.180992659 | 0.01558059 | 1.54 | 1.66 |
| *Col5a2* | -1.184304596 | 3.14E-05 | 1.77 | 1.77 |
| *Marcksl1* | -1.186945722 | 1.95E-06 | 1.69 | 1.69 |
| *Axl* | -1.186955146 | 4.01E-05 | 1.18 | 1.18 |
| *Cpxm1* | -1.188348726 | 0.00090531 | 0.02 | 1.08 |
| *Lims2* | -1.189954355 | 0.00184079 | 1.95 | 1.95 |
| *Prkcdbp* | -1.192897226 | 1.03E-07 | 1.53 | 2.05 |
| *Ier5l* | -1.199328748 | 0.00255369 | 0.39 | 2.46 |
| *St6galnac2* | -1.203263539 | 0.00072898 | 1.1 | 1.1 |
| *Ntrk3* | -1.205610158 | 0.02441645 | 0.92 | 0.92 |
| *Sema3b* | -1.215098759 | 0.00292373 | 1.34 | 1.34 |
| *Slc28a2* | -1.217725326 | 0.00403673 | 1.77 | 1.77 |
| *Itgb3* | -1.232703642 | 0.00178878 | -0.25 | 1.5 |
| *Fkbp10* | -1.233567526 | 1.53E-06 | 2 | 2 |
| *Tead3* | -1.234505189 | 4.77E-05 | 0.11 | 1.16 |
| *Cdk6* | -1.237527225 | 0.00016859 | 0.98 | 0.98 |
| *Slc35f1* | -1.242972178 | 0.03137184 | 1.21 | 1.21 |
| *Bdkrb2* | -1.244379936 | 0.00120875 | 1.58 | 1.64 |
| *Tgfbr2* | -1.245010873 | 4.09E-06 | 0.28 | 1.65 |
| *Tll1* | -1.24588073 | 0.0004173 | 0.75 | 0.75 |
| *Opcml* | -1.247961492 | 0.04103966 | 0.92 | 0.92 |
| *Zfp385d* | -1.250103588 | 0.03260237 | 1.19 | 1.19 |
| *Pear1* | -1.250360332 | 1.79E-05 | 1.61 | 1.76 |
| *Dnase2* | -1.251288788 | 6.08E-07 | 0.23 | 1.15 |
| *Lrrc8c* | -1.252752743 | 0.00565922 | 1.53 | 1.53 |
| *Crim1* | -1.276710245 | 8.65E-06 | -0.02 | 1.51 |
| *Chrm4* | -1.287600273 | 0.02972132 | 1.22 | 1.66 |

| | | | | |
|---|---|---|---|---|
| *Sfrp1* | -1.291672443 | 0.00051676 | 0.93 | 2.39 |
| *Zfpm2* | -1.291995961 | 0.00124334 | 1.56 | 1.57 |
| *Pde3a* | -1.300721083 | 0.00011806 | 1.11 | 1.11 |
| *Creb5* | -1.304224817 | 0.0140712 | 1.51 | 1.85 |
| *Slc22a18* | -1.306976196 | 0.0098599 | 1.89 | 1.89 |
| *Plau* | -1.308381866 | 0.00027571 | 1.25 | 1.25 |
| *Ptprv* | -1.311692073 | 0.00920613 | 1.56 | 1.57 |
| *Penk* | -1.312441457 | 0.00878525 | 1.1 | 1.1 |
| *Tril* | -1.314580504 | 2.37E-06 | 1.23 | 1.23 |
| *Ebf3* | -1.332153012 | 0.00012622 | 1.02 | 1.12 |
| *Aebp1* | -1.344620389 | 1.91E-06 | 1.52 | 1.78 |
| *Fgf2* | -1.349442482 | 0.03565955 | 1.09 | 1.09 |
| *Synpo* | -1.358265831 | 0.00040483 | 1.77 | 1.77 |
| *Kazn* | -1.370355451 | 0.00091925 | 0.86 | 1.04 |
| *Gpnmb* | -1.372427435 | 0.01654222 | 1.25 | 1.25 |
| *Trpv4* | -1.375485228 | 0.00430153 | 0.78 | 0.78 |
| *Fbxl7* | -1.383319865 | 0.03487334 | 1.25 | 1.25 |
| *Slit2* | -1.399880308 | 1.03E-07 | 1.16 | 1.35 |
| *Kcne4* | -1.411781343 | 0.00013455 | 1.45 | 1.45 |
| *Nbl1* | -1.412824083 | 8.04E-09 | 1.94 | 1.94 |
| *Npr1* | -1.416383357 | 8.76E-05 | 0.78 | 0.78 |
| *Gpr4* | -1.417968664 | 0.01301537 | 1.05 | 1.05 |
| *Begain* | -1.424464057 | 0.04062812 | -0.11 | 1.14 |
| *Kcns3* | -1.424644786 | 0.00116292 | 1.1 | 1.12 |
| *Ovol1* | -1.429615073 | 0.00597205 | -0.11 | 1.06 |
| *Rem1* | -1.431477374 | 0.00020339 | 1.28 | 1.28 |
| *Sox17* | -1.433141964 | 9.99E-06 | 1.43 | 1.43 |
| *Tbx3* | -1.434908248 | 0.00062297 | 1.37 | 1.37 |
| *Anxa2* | -1.439108233 | 8.53E-06 | 1.46 | 1.46 |
| *Osr1* | -1.440370649 | 0.00072836 | 1.45 | 1.51 |
| *Rgs6* | -1.460510165 | 0.0012388 | 1.47 | 1.47 |
| *Ebf1* | -1.463904911 | 8.85E-06 | 1.07 | 1.14 |
| *Foxc1* | -1.470367595 | 0.01172413 | 1.18 | 1.18 |
| *Maf* | -1.471174313 | 0.00161237 | 0.92 | 0.92 |
| *Vwf* | -1.473760749 | 0.0007908 | 1.87 | 1.87 |
| *Hba2* | -1.474037864 | 0.00382348 | 0.82 | 0.82 |
| *Cldn5* | -1.476774291 | 0.00039329 | 1.15 | 1.16 |
| *Sema3g* | -1.479428317 | 3.75E-06 | 1.05 | 2.29 |
| *Gli3* | -1.487920011 | 0.00123646 | 1.4 | 1.4 |
| *Gpihbp1* | -1.48922349 | 6.40E-06 | 1.67 | 1.67 |
| *Madcam1* | -1.493166081 | 0.0005662 | 0.52 | 1.28 |
| *Tal1* | -1.498100093 | 0.00807301 | 1.29 | 1.45 |
| *Fbln1* | -1.502482364 | 1.70E-12 | 1.29 | 1.29 |
| *Cybrd1* | -1.510727632 | 2.90E-05 | 1.12 | 1.12 |
| *Col6a2* | -1.51282244 | 1.42E-07 | 1.05 | 1.05 |

| | | | | |
|---|---|---|---|---|
| *Hba1* | -1.513007131 | 0.00293138 | 0.82 | 0.82 |
| *Olfml2b* | -1.522551914 | 4.45E-07 | 1.84 | 1.84 |
| *Vipr1* | -1.526789128 | 0.0055554 | -0.25 | 1.38 |
| *Igf2bp1* | -1.528908077 | 0.04615973 | 1.14 | 1.14 |
| *Crispld2* | -1.538684083 | 1.46E-05 | 1.74 | 2.07 |
| *Klhl29* | -1.540076572 | 0.00013888 | 0.09 | 0.99 |
| *Gng8* | -1.541877728 | 0.04151359 | 1.2 | 1.2 |
| *Fabp5* | -1.549056194 | 6.81E-06 | 0.53 | 1.81 |
| *Ldb2* | -1.552164142 | 6.84E-10 | 1.47 | 1.47 |
| *Slit3* | -1.557113938 | 1.68E-07 | 1.2 | 1.2 |
| *Nrxn2* | -1.55933862 | 0.0310203 | -0.34 | 0.93 |
| *Osr2* | -1.560454836 | 0.01515636 | 1.21 | 1.21 |
| *Ptgir* | -1.565343232 | 0.00056337 | 0.26 | 1.38 |
| *S100a6* | -1.574912993 | 7.22E-09 | 1.12 | 1.12 |
| *Prrx1* | -1.576589637 | 2.71E-05 | 1.36 | 1.57 |
| *Col5a1* | -1.577389507 | 4.65E-07 | 0.32 | 1.4 |
| *Chit1* | -1.592265479 | 0.00964426 | 1.52 | 1.52 |
| *Kif5a* | -1.593576394 | 0.00473464 | 1.18 | 1.39 |
| *Map3k6* | -1.603198516 | 5.63E-05 | 0.18 | 1.42 |
| *Cd248* | -1.608271695 | 0.00013888 | 1.51 | 1.51 |
| *Ddit4* | -1.62475095 | 6.15E-12 | 2.04 | 2.04 |
| *Dnm3* | -1.625608499 | 2.99E-05 | 0.51 | 0.51 |
| *Fbln5* | -1.627040032 | 2.61E-09 | 1.41 | 1.41 |
| *Aldh3a1* | -1.630794834 | 0.024019 | 2.06 | 2.06 |
| *Aldh1a3* | -1.639723645 | 0.00195836 | -0.12 | 0.95 |
| *Rassf4* | -1.640247533 | 4.27E-06 | 1.25 | 1.25 |
| *Fbn1* | -1.645412773 | 9.48E-07 | 0.09 | 0.92 |
| *Fosb* | -1.653652909 | 0.00070622 | 1.19 | 1.2 |
| *Pla2g5* | -1.664557742 | 0.0223172 | 2.37 | 2.37 |
| *Apoe* | -1.665045018 | 4.07E-05 | 1.71 | 1.71 |
| *Aplnr* | -1.669045952 | 5.10E-11 | 2.15 | 2.15 |
| *Myocd* | -1.689240689 | 0.0352891 | 1.05 | 1.05 |
| *Acss1* | -1.69164178 | 9.13E-10 | 1.79 | 1.79 |
| *Rab6b* | -1.693048187 | 8.78E-05 | 1.71 | 1.71 |
| *Chp2* | -1.696630517 | 5.02E-05 | 1.21 | 1.21 |
| *St14* | -1.698004068 | 2.06E-08 | -0.25 | 1.32 |
| *Aldh1a2* | -1.700963545 | 2.91E-06 | 1.38 | 1.38 |
| *Ebf2* | -1.704649605 | 1.20E-05 | 1.1 | 1.33 |
| *Ablim3* | -1.715096347 | 1.78E-06 | 1.51 | 1.51 |
| *Shank3* | -1.722576488 | 2.33E-07 | 1.03 | 1.05 |
| *Osm* | -1.728332031 | 0.0151486 | 1.93 | 1.93 |
| *Cdh3* | -1.72880026 | 3.06E-10 | 1.31 | 1.31 |
| *Lgals3* | -1.732769431 | 4.26E-11 | 1.53 | 1.53 |
| *Ccdc80* | -1.736729959 | 5.61E-07 | 1.17 | 1.17 |
| *Doc2b* | -1.740608461 | 0.00056591 | 1.83 | 2.43 |

| | | | | |
|---|---|---|---|---|
| *Cyp1b1* | -1.741808932 | 0.04872477 | 0.76 | 0.76 |
| *Spon1* | -1.750471052 | 4.60E-10 | 1.29 | 1.29 |
| *Hoxd9* | -1.751751715 | 0.04017923 | 1.55 | 1.55 |
| *Pmp22* | -1.754321312 | 7.07E-12 | -0.15 | 1.11 |
| *Plaur* | -1.771617084 | 0.00268887 | 1.24 | 1.24 |
| *Akap5* | -1.771772317 | 0.03025533 | 1.21 | 1.21 |
| *Tcf15* | -1.793043006 | 0.00123452 | 1.69 | 2.11 |
| *Notch3* | -1.796400813 | 0.00011138 | 0.12 | 1.52 |
| *Mrvi1* | -1.80813096 | 2.65E-08 | 2.27 | 2.62 |
| *Tph1* | -1.818756232 | 0.03783181 | 0.99 | 0.99 |
| *Gzmb* | -1.829527933 | 0.01298498 | 1.41 | 1.41 |
| *Duox1* | -1.835882635 | 0.00558628 | 1.26 | 1.26 |
| *Slc26a10* | -1.844677161 | 9.06E-06 | 0.64 | 1.12 |
| *Tnfsf9* | -1.844786318 | 0.02105566 | 1.11 | 1.11 |
| *Fam107a* | -1.867252201 | 7.99E-05 | 1.35 | 1.35 |
| *Cdh13* | -1.884017447 | 2.75E-10 | 0.82 | 0.82 |
| *Nat8l* | -1.891086057 | 0.00332645 | 0.99 | 0.99 |
| *Duoxa1* | -1.905516136 | 0.00152481 | 1.26 | 1.26 |
| *Cebpd* | -1.909057446 | 1.27E-07 | 2.58 | 2.58 |
| *Fam129a* | -1.961680569 | 3.61E-15 | 1.14 | 1.14 |
| *Prima1* | -1.965318198 | 0.0052549 | 1.65 | 1.65 |
| *Galnt16* | -1.979299393 | 2.91E-08 | -0.19 | 1.01 |
| *Grifin* | -1.985107296 | 0.01187573 | 1.2 | 1.2 |
| *Krt75* | -2.001554883 | 0.0361666 | 1.08 | 1.11 |
| *Col5a3* | -2.014787952 | 9.96E-11 | 0.29 | 1.5 |
| *Tgfbr3* | -2.025772465 | 1.23E-09 | 1.75 | 1.97 |
| *Aif1l* | -2.031047695 | 8.29E-09 | 1.99 | 1.99 |
| *Lrg1* | -2.043965964 | 7.09E-08 | -0.01 | 1.14 |
| *Notum* | -2.066472405 | 0.015563 | -0.15 | 1.23 |
| *Scnn1g* | -2.089601282 | 0.00254228 | 0.14 | 1.52 |
| *Cyp26b1* | -2.092298438 | 6.43E-05 | 1.24 | 1.24 |
| *Faim2* | -2.093213479 | 0.0353093 | -0.03 | 1.61 |
| *Tnf* | -2.101881623 | 0.00201979 | 1.01 | 1.02 |
| *Hrh1* | -2.102717612 | 0.00537845 | 1.63 | 1.63 |
| *Rgma* | -2.109601452 | 4.87E-05 | 1.27 | 1.42 |
| *Mcpt10* | -2.110335309 | 0.01926131 | 1.97 | 2.01 |
| *LOC257642* | -2.116441928 | 0.00378225 | 2.37 | 2.91 |
| *Fmo1* | -2.141187463 | 0.01557725 | 1.32 | 1.32 |
| *Cygb* | -2.148865372 | 5.92E-13 | 1.27 | 1.51 |
| *C2* | -2.161004656 | 4.48E-05 | 1.44 | 1.44 |
| *Thbd* | -2.162644472 | 2.77E-10 | 0.94 | 0.94 |
| *Mcemp1* | -2.164210744 | 0.02691981 | 0.11 | 1.33 |
| *Cmtm5* | -2.192103779 | 0.00445493 | 1.8 | 1.8 |
| *Lrrc32* | -2.225116362 | 1.98E-08 | 0.93 | 0.93 |
| *C4b* | -2.225416056 | 0.00017313 | 2.34 | 2.34 |

| | | | | |
|---|---|---|---|---|
| *RGD1310587* | -2.235801864 | 8.04E-16 | 1.31 | 1.31 |
| *Dusp27* | -2.24798733 | 0.01751157 | 1.48 | 1.48 |
| *Sox7* | -2.279651528 | 2.72E-10 | 1.06 | 1.06 |
| *Nr4a3* | -2.297921651 | 0.00016048 | 1.94 | 1.94 |
| *Fam181b* | -2.315466706 | 0.03687988 | 1.32 | 1.32 |
| *Olr837* | -2.325087785 | 0.02668655 | 1.39 | 1.39 |
| *Nrgn* | -2.37482875 | 0.00084199 | 1.05 | 1.05 |
| *Rbp7* | -2.400233377 | 0.00113014 | 1.84 | 1.84 |
| *Acsbg1* | -2.417826202 | 0.00585688 | 1.81 | 1.82 |
| *Sstr4* | -2.42153914 | 0.01640004 | 1.08 | 1.08 |
| *Icam5* | -2.453665289 | 0.00371063 | 1.13 | 1.42 |
| *Alox5* | -2.491578193 | 0.00010677 | 0.99 | 1.06 |
| *Mpz* | -2.493595543 | 0.00267129 | 0.42 | 1.17 |
| *Cxcr2* | -2.494912085 | 0.01269533 | 1.04 | 1.09 |
| *Qsox1* | -2.498825371 | 4.14E-10 | 1.17 | 1.17 |
| *Rspo2* | -2.542645744 | 0.00253528 | 1.07 | 1.07 |
| *Bcat1* | -2.542921326 | 1.88E-06 | 0.92 | 0.92 |
| *Prph* | -2.638463 | 2.31E-09 | 0.43 | 1.32 |
| *Lrrc3* | -2.687130609 | 6.42E-06 | 1.07 | 1.11 |
| *Il10* | -2.74056139 | 0.00253942 | 1.27 | 1.27 |
| *Mt1* | -2.800206934 | 1.57E-11 | 1.36 | 1.36 |
| *Igfbp5* | -2.809571507 | 4.27E-17 | 1.69 | 1.69 |
| *Chrna7* | -2.897271891 | 0.00304632 | 0.47 | 1.82 |
| *Krt17* | -2.903026701 | 0.00018014 | 1.04 | 1.14 |
| *Col1a1* | -2.98651527 | 2.44E-14 | 1.31 | 1.38 |
| *Prrx2* | -2.990548652 | 3.69E-06 | 1.37 | 1.37 |
| *Grm7* | -3.009016858 | 0.00292026 | 0.8 | 0.8 |
| *Chat* | -3.030143782 | 6.26E-08 | 1.05 | 1.36 |
| *Bdkrb1* | -3.051653833 | 0.00374895 | 1.51 | 1.52 |
| *LOC299282* | -3.09073636 | 0.00445567 | 1.61 | 1.61 |
| *Icam4* | -3.129070676 | 0.00398866 | 1.13 | 1.42 |
| *Bmper* | -3.143386134 | 0.00094311 | 1.52 | 1.52 |
| *Aqp7* | -3.158698026 | 0.01174187 | 1.51 | 1.51 |
| *Tmeff2* | -3.192916277 | 0.00025671 | 1.09 | 1.09 |
| *Prr15* | -3.221379912 | 8.72E-08 | -0.22 | 1 |
| *Tpsb2* | -3.228519828 | 0.00261419 | 0.65 | 1.54 |
| *Serpina3n* | -3.262900737 | 0.00022644 | 1.7 | 1.7 |
| *Krt14* | -3.308057617 | 0.00351853 | 1.21 | 1.21 |
| *Tac3* | -3.335851407 | 0.0001784 | 0.41 | 1.16 |
| *Padi4* | -3.361630865 | 1.74E-07 | 1.35 | 1.39 |
| *Nos2* | -3.393053129 | 2.80E-07 | 1.35 | 1.35 |
| *Cma1* | -3.461227959 | 0.00170959 | 2.29 | 2.3 |
| *Cnga3* | -3.522698493 | 0.02279103 | -0.03 | 1.24 |
| *Wt1* | -3.533936452 | 1.46E-08 | 1.08 | 1.25 |
| *Sfrp4* | -3.539032099 | 2.42E-11 | 1.34 | 1.34 |

| | | | | |
|---|---|---|---|---|
| *Cxcl14* | -3.597216658 | 6.51E-13 | 0.96 | 0.96 |
| *Gpc5* | -3.617546985 | 0.01371774 | -0.03 | 1.3 |
| *Dleu7* | -3.622316911 | 0.01386764 | 0.69 | 0.69 |
| *Tbx18* | -3.703076344 | 0.0021956 | 1.1 | 1.1 |
| *Upk1b* | -3.775238805 | 1.34E-05 | 0.58 | 1.78 |
| *Lamc3* | -3.843259855 | 1.27E-12 | 1.49 | 1.49 |
| *Mcpt1l1* | -3.890313117 | 0.00114653 | 2.29 | 2.3 |
| *Igfbp6* | -3.902829408 | 3.20E-17 | 0.07 | 1.18 |
| *Nkain4* | -4.308001261 | 2.79E-08 | -0.29 | 1.07 |
| *Chrdl2* | -4.511761861 | 4.46E-06 | 0.94 | 0.94 |
| *Cxcl3* | -4.578520208 | 0.04122249 | 1.36 | 1.36 |
| *Wfdc18* | -4.605598761 | 0.00968292 | 0.95 | 0.95 |
| *Nos1* | -4.685240331 | 3.31E-09 | 2.03 | 2.24 |
| *Prkg2* | -4.78225845 | 6.67E-05 | 1.29 | 1.29 |
| *Rgs20* | -4.814869997 | 0.02508581 | 1.14 | 1.14 |
| *Olr1750* | -4.937714239 | 0.02154104 | 1.17 | 1.17 |
| *Krt72* | -5.160743698 | 0.00536024 | 1.34 | 1.36 |
| *Nog* | -5.399373298 | 0.00336677 | 1.33 | 1.33 |
| *Tlx2* | -5.520456828 | 0.00163957 | 1.51 | 1.67 |
| *Mylk2* | -6.296179471 | 2.45E-15 | 1.11 | 1.11 |

**anges**

**Supplemental Table S5 Differentially Expressed Genes with Consistent H3K27Ac Histone Mark Ch**

**2-wk Islets**

| Gene | RNAseq_logFC | RNAseq_FDR | H3K27Ac_avgpeak_logFC | H3K27Ac_maxpeak_logFC |
|---|---|---|---|---|
| *Gpd1* | 1.881588907 | 0.00959853 | 0.87 | 0.91 |
| *Osm* | 1.770787462 | 0.02244283 | 0.92 | 0.92 |
| *Cidec* | 1.618750565 | 0.04384976 | 0.88 | 0.88 |
| *Csrnp1* | 1.133493577 | 0.01165654 | 0.47 | 0.49 |
| *Cmklr1* | 1.095010889 | 0.01822828 | 0.86 | 0.86 |
| *Anxa2* | 0.992594856 | 0.01197936 | 0.69 | 1 |
| *Tmem229a* | 0.945605605 | 0.03778154 | 0.92 | 0.92 |
| *Coq10b* | 0.942036679 | 0.00491713 | 0.89 | 0.89 |
| *Itgb3* | 0.929352943 | 0.0414565 | 1.11 | 1.11 |
| *Fam46a* | 0.927059372 | 0.01440697 | 0.49 | 0.49 |
| *Dgat2* | 0.914704136 | 0.01637179 | 0.64 | 0.64 |
| *RGD1311946* | 0.896859722 | 0.01037667 | 0.52 | 0.52 |
| *Ppp1r3b* | 0.886573108 | 0.03949695 | 0.44 | 0.44 |
| *Slc25a25* | 0.878995218 | 0.00814116 | 0.86 | 0.86 |
| *Vim* | 0.868352027 | 0.03971334 | 0.09 | 0.09 |
| *Pcolce* | 0.865457524 | 0.00742609 | 1.12 | 1.12 |
| *Cdr2* | 0.833761146 | 0.0005379 | 1.48 | 1.48 |
| *Rcan1* | 0.819370015 | 0.00916558 | 0.83 | 1.07 |
| *Id3* | 0.818937438 | 0.01770793 | 0.44 | 0.44 |
| *S100a10* | 0.816245477 | 0.00810099 | 0.76 | 0.91 |
| *Epha2* | 0.798677553 | 0.02856194 | 0.62 | 0.75 |
| *Fbln1* | 0.792629516 | 0.00149055 | 0.86 | 0.9 |
| *Ckb* | 0.78502191 | 0.00068103 | 0.3 | 0.81 |
| *Klf6* | 0.784367992 | 0.02207777 | 0.28 | 0.28 |
| *Nes* | 0.773566927 | 0.00591614 | 0.87 | 0.87 |
| *Prkcdbp* | 0.755397962 | 0.00483877 | 0.37 | 0.37 |
| *Pdlim1* | 0.746919946 | 0.00143175 | 0.61 | 0.91 |
| *Dse* | 0.709336586 | 0.0450081 | 0.28 | 0.28 |
| *Rab31* | 0.691366043 | 0.02153968 | 0.73 | 0.75 |
| *Trib2* | 0.685050163 | 0.0424242 | 0.42 | 0.76 |
| *Sowahc* | 0.675172421 | 0.01424013 | 0.14 | 0.14 |
| *Mgat3* | 0.657525452 | 0.01549967 | 1.1 | 1.1 |
| *Sdc4* | 0.649163445 | 0.01440697 | 0.67 | 0.67 |
| *Myl12a* | 0.642437702 | 0.00587165 | 0.46 | 0.46 |
| *B4galt5* | 0.629756646 | 0.0255003 | 0.61 | 0.61 |
| *Rbpms* | 0.629447508 | 0.03349242 | 0.04 | 0.04 |
| *Tnfrsf1a* | 0.618671252 | 0.02916048 | 0.74 | 0.74 |
| *Hsd17b12* | 0.572503572 | 0.00784753 | 0.33 | 0.33 |
| *Cnpy4* | 0.571850256 | 0.03280891 | 0.28 | 0.28 |
| *Mfhas1* | 0.569986743 | 0.04439975 | 0.13 | 0.13 |
| *Ddah2* | 0.547065643 | 0.04211705 | 0.67 | 0.67 |

| | | | | |
|---|---|---|---|---|
| *Itsn1* | 0.541657129 | 0.04769307 | 0.44 | 0.44 |
| *Spry1* | 0.529459325 | 0.04529198 | 0.32 | 0.32 |
| *Arpc4* | 0.527135311 | 0.0110667 | 0.08 | 0.08 |
| *P4ha1* | 0.517231816 | 0.02555594 | 0.17 | 0.17 |
| *Cycs* | 0.514237938 | 0.04342333 | 1.19 | 1.19 |
| *Cdc42ep3* | 0.502912615 | 0.0497978 | 0.12 | 0.12 |
| *Sec23a* | 0.500900426 | 0.02399984 | 0.25 | 0.25 |
| *Capn2* | 0.497802675 | 0.04413315 | 0.56 | 0.56 |
| *Snx18* | 0.492144506 | 0.03303398 | 0.43 | 0.45 |
| *Hif1a* | 0.484104552 | 0.03017262 | 0.37 | 0.37 |
| *Ywhah* | 0.448264711 | 0.04852767 | 0.19 | 0.19 |
| *Mapk8ip3* | -0.49155432 | 0.04608431 | -0.77 | -0.77 |
| *Phf1* | -0.499781038 | 0.03640908 | -0.4 | -0.4 |
| *Nfxl1* | -0.512210392 | 0.0374073 | -0.64 | -0.64 |
| *Gtf3c2* | -0.512226642 | 0.00818887 | -0.86 | -0.86 |
| *Ppan* | -0.513132188 | 0.04833756 | -0.35 | -0.45 |
| *Pick1* | -0.529275459 | 0.04951297 | -0.39 | -0.39 |
| *Atp13a1* | -0.530985251 | 0.02676731 | -0.76 | -0.88 |
| *Nob1* | -0.536647583 | 0.02477065 | -0.4 | -0.4 |
| *Trit1* | -0.547825712 | 0.01392351 | -0.25 | -0.25 |
| *Tada2a* | -0.548507658 | 0.04114461 | -0.56 | -0.57 |
| *Aup1* | -0.552856799 | 0.03507198 | -0.46 | -0.46 |
| *Zfp142* | -0.566008339 | 0.03986618 | -0.25 | -0.29 |
| *Trmt1* | -0.577306825 | 0.01030193 | -0.4 | -0.64 |
| *Igsf11* | -0.584956315 | 0.02638313 | -0.84 | -0.84 |
| *Dcaf11* | -0.58993784 | 0.00988277 | -0.06 | -0.29 |
| *Ercc6* | -0.595413541 | 0.00694755 | -0.32 | -0.32 |
| *Acd* | -0.601179352 | 0.04681118 | -0.48 | -0.79 |
| *Cherp* | -0.601379391 | 0.04350988 | -0.68 | -0.68 |
| *Tnip2* | -0.608886373 | 0.03889433 | -0.55 | -0.55 |
| *Tmem214* | -0.628035983 | 0.04162073 | -0.44 | -0.5 |
| *Atg13* | -0.633234936 | 0.00885295 | -0.44 | -0.44 |
| *Rundc3b* | -0.633344018 | 0.0351456 | -0.33 | -0.33 |
| *Srp68* | -0.640554893 | 0.01807594 | -0.86 | -1.11 |
| *Ccnl2* | -0.640617188 | 0.0007914 | -0.46 | -0.46 |
| *Snhg11* | -0.654692157 | 0.04466069 | -0.35 | -0.35 |
| *Vars2* | -0.67395121 | 0.01346001 | -0.34 | -0.34 |
| *Akap8l* | -0.685436588 | 0.00030368 | -0.1 | -0.37 |
| *Isyna1* | -0.68656464 | 0.01382367 | -0.72 | -0.8 |
| *Pck2* | -0.688530259 | 0.02099762 | -0.49 | -0.49 |
| *Rtkn* | -0.690769167 | 0.00973712 | -0.47 | -0.48 |
| *Esrp1* | -0.694801442 | 0.01535867 | -0.6 | -0.6 |
| *Arfip2* | -0.695716249 | 0.01252019 | -0.42 | -0.49 |
| *Camkk2* | -0.705812521 | 0.0338979 | -0.5 | -0.5 |
| *Dhrs1* | -0.71545108 | 0.03627033 | -0.45 | -0.45 |

| | | | | |
|---|---|---|---|---|
| *Mlxipl* | -0.724825394 | 0.01640628 | -0.71 | -0.76 |
| *Slc44a3* | -0.735895003 | 0.01036929 | -0.45 | -0.45 |
| *Klhdc8a* | -0.767479944 | 0.04108371 | -0.41 | -0.41 |
| *Tmem184a* | -0.77787278 | 0.00891512 | -0.51 | -0.51 |
| *Angptl6* | -0.779178918 | 0.01861843 | -0.35 | -0.45 |
| *Angel1* | -0.781365387 | 0.01193046 | -0.6 | -0.6 |
| *Tanc2* | -0.789130407 | 0.00076594 | -0.19 | -0.19 |
| *Slc7a1* | -0.794058155 | 0.02156974 | -0.55 | -0.55 |
| *Clmn* | -0.806639705 | 0.04391289 | -0.25 | -0.25 |
| *Dhrs4* | -0.828308465 | 0.00512655 | -0.57 | -0.58 |
| *Krtcap2* | -0.839188584 | 0.04035278 | -0.66 | -0.66 |
| *Zmynd10* | -0.83986015 | 0.03627033 | -0.86 | -0.86 |
| *Abcc10* | -0.862413453 | 0.01009017 | -0.13 | -0.13 |
| *Dqx1* | -0.868308613 | 0.00353822 | -0.46 | -0.46 |
| *Plk5* | -0.86970536 | 0.0391428 | -0.56 | -0.57 |
| *Nt5dc3* | -0.871043278 | 0.00912202 | -0.24 | -0.47 |
| *Trabd* | -0.882136368 | 0.02320154 | -0.49 | -0.54 |
| *Hm13* | -0.889206654 | 0.01844836 | -0.56 | -0.56 |
| *Pde4c* | -0.89434459 | 0.03414726 | -0.46 | -0.68 |
| *Ccdc62* | -0.896821239 | 0.0005473 | -0.24 | -0.24 |
| *Reep5* | -0.915447015 | 0.04487622 | -0.41 | -0.41 |
| *Wdtc1* | -0.916726251 | 0.002976 | -1.01 | -1.01 |
| *Pan2* | -0.923168819 | 4.63E-06 | -0.57 | -0.58 |
| *Pdcd4* | -0.975128477 | 0.00653472 | -0.13 | -0.13 |
| *Slc7a8* | -0.975234484 | 0.0007097 | -0.19 | -0.19 |
| *Foxa3* | -0.977232581 | 0.03162049 | -0.58 | -0.67 |
| *Arhgap8* | -0.983207722 | 0.00437266 | -0.49 | -0.49 |
| *Cyp2t1* | -0.990153264 | 0.01140678 | -0.63 | -0.63 |
| *Derl3* | -1.007779627 | 0.0391428 | -0.56 | -0.57 |
| *Rnpepl1* | -1.031389413 | 0.00116886 | -0.64 | -0.64 |
| *Ulk1* | -1.031906416 | 2.87E-05 | -0.41 | -0.41 |
| *Gga1* | -1.063106726 | 0.00013843 | -0.41 | -0.41 |
| *Bcat2* | -1.083988051 | 0.01301792 | -0.65 | -0.98 |
| *Spag8* | -1.092973446 | 0.0060326 | -0.19 | -0.19 |
| *Mast3* | -1.104643885 | 0.00026894 | -0.48 | -0.56 |
| *Psat1* | -1.132231266 | 0.0053876 | -0.13 | -0.13 |
| *Xbp1* | -1.138901562 | 0.01637348 | -0.41 | -0.56 |
| *Pde4a* | -1.147068126 | 0.01103483 | -0.53 | -0.53 |
| *Rnh1* | -1.148963498 | 0.01392351 | -0.28 | -0.28 |
| *Hdac11* | -1.228258452 | 7.32E-05 | -0.2 | -0.2 |
| *Tst* | -1.255004826 | 0.01411028 | -0.07 | -0.14 |
| *Slc11a2* | -1.290849464 | 0.00022634 | -0.34 | -0.34 |
| *Macrod1* | -1.292606444 | 0.00056658 | -0.49 | -0.49 |
| *Slc25a23* | -1.297882141 | 2.41E-05 | -0.52 | -0.52 |
| *Slc4a8* | -1.338425044 | 0.0040263 | -0.7 | -0.7 |

| | | | | |
|---|---|---|---|---|
| *Ppard* | -1.358515446 | 5.15E-05 | -0.06 | -0.06 |
| *Trim46* | -1.379548433 | 0.0005379 | -0.66 | -0.66 |
| *Gpt2* | -1.390236223 | 0.00028318 | -0.57 | -0.59 |
| *Sfxn2* | -1.416253557 | 0.00233662 | -0.51 | -0.51 |
| *Dram1* | -1.418617122 | 0.00140878 | -0.12 | -0.12 |
| *Kirrel2* | -1.430126527 | 0.02384424 | -0.86 | -0.86 |
| *RGD1309139* | -1.514243567 | 0.01055967 | -0.24 | -0.24 |
| *Slc22a4* | -1.527437172 | 0.01698386 | -0.59 | -0.59 |
| *Aldh1l2* | -1.542084404 | 0.00587165 | -0.75 | -0.75 |
| *Fkbp11* | -1.547670011 | 0.00060019 | -0.6 | -0.6 |
| *Sel1l* | -1.605428539 | 0.00383921 | -0.38 | -0.38 |
| *Pabpc4* | -1.659158786 | 2.16E-05 | -0.52 | -0.52 |
| *Pycr1* | -1.849554707 | 0.00018243 | -0.68 | -0.87 |
| *Slc41a1* | -1.895711894 | 7.32E-05 | -0.14 | -0.14 |
| *Muc1* | -2.077133013 | 0.00469833 | -0.2 | -0.2 |
| *RGD1305928* | -2.104395175 | 0.00353901 | -0.33 | -0.33 |
| *Bhlha15* | -2.118106559 | 8.77E-05 | -0.66 | -0.66 |

**10-wk Islets**

| Gene | RNAseq_logFC | RNAseq_FDR | H3K27Ac_avgpeak_logFC | H3K27Ac_maxpeak_logFC |
|---|---|---|---|---|
| *Mir495* | 5.374616315 | 0.00350455 | 1.09 | 1.09 |
| *Mir758* | 1.503425572 | 0.02564589 | 1.09 | 1.09 |
| *Kcnk2* | 1.126994395 | 0.0218037 | 1.11 | 1.11 |
| *Fbxo2* | 0.998585212 | 0.01142895 | 0.95 | 0.95 |
| *Hap1* | 0.987419561 | 0.0003022 | 0.85 | 0.85 |
| *Oas1f* | 0.945701095 | 0.04097713 | 0.91 | 0.91 |
| *Mdh1* | 0.878683784 | 0.00147035 | 0.7 | 0.7 |
| *Wdpcp* | 0.873272817 | 1.10E-05 | 0.7 | 0.7 |
| *Gcgr* | 0.859937047 | 0.01876891 | 0.81 | 0.81 |
| *Tmem150c* | 0.850662811 | 0.00308922 | 0.83 | 0.83 |
| *Trpm5* | 0.830189818 | 0.02145042 | 0.96 | 0.96 |
| *Col16a1* | 0.812785853 | 0.0002585 | 0.89 | 0.89 |
| *Gpr6* | 0.791913855 | 0.03789497 | 1 | 1 |
| *Arc* | 0.788418732 | 0.03891885 | 0.83 | 0.83 |
| *Camk1g* | 0.689385202 | 0.02999237 | 0.52 | 0.52 |
| *Ndrg4* | 0.632373711 | 0.0497661 | 1.01 | 1.1 |
| *Nme3* | 0.629972319 | 0.00319567 | 0.79 | 0.79 |
| *Dnpep* | 0.627657821 | 0.00304082 | 1.17 | 1.17 |
| *Vegfa* | 0.562409983 | 0.02351337 | 0.79 | 0.79 |
| *Fbxo44* | 0.562134852 | 0.03713837 | 0.95 | 0.95 |
| *Atp6v0e2* | 0.558255482 | 0.00413979 | 0.91 | 0.91 |
| *Rab3ip* | 0.557358668 | 0.00916433 | 1.01 | 1.01 |
| *Repin1* | 0.529061147 | 0.04217907 | 1.09 | 1.35 |
| *Slc26a2* | 0.519523952 | 0.01654222 | 1.2 | 1.2 |
| *Fam216a* | 0.51846525 | 0.02424808 | 0.8 | 0.8 |

| | | | | |
|---|---|---|---|---|
| *Pld3* | 0.503574592 | 0.02033336 | 1.46 | 1.46 |
| *Tfam* | 0.501433582 | 0.01479307 | 1.1 | 1.1 |
| *P4ha2* | 0.479030806 | 0.01566925 | 0.98 | 0.98 |
| *Mcf2l* | 0.466933434 | 0.05002442 | 1.19 | 1.19 |
| *Anxa11* | -0.410540362 | 0.03783181 | -0.7 | -0.7 |
| *Camk2d* | -0.413516362 | 0.04335305 | -0.88 | -0.88 |
| *Rgl2* | -0.435964668 | 0.04672833 | -0.71 | -0.71 |
| *Uap1* | -0.447031123 | 0.04934185 | -0.98 | -1.03 |
| *Wbp1l* | -0.4505607 | 0.02025392 | -0.75 | -0.75 |
| *Sft2d2* | -0.450648429 | 0.03783181 | -0.81 | -0.81 |
| *Jup* | -0.457002166 | 0.02033336 | -0.69 | -0.69 |
| *Efhd2* | -0.463030626 | 0.04110173 | -0.52 | -0.52 |
| *Oxa1l* | -0.471955106 | 0.01991716 | -0.61 | -0.61 |
| *Dusp6* | -0.482828618 | 0.04605076 | -0.63 | -0.63 |
| *Eif1* | -0.485321531 | 0.02371717 | -0.81 | -0.81 |
| *Eif3h* | -0.49051131 | 0.03769312 | -0.79 | -0.79 |
| *Zfp598* | -0.495096061 | 0.04800199 | -1.06 | -1.34 |
| *Map3k11* | -0.495308473 | 0.02226293 | -0.84 | -0.84 |
| *Myl12a* | -0.505895528 | 0.02183961 | -0.74 | -0.74 |
| *Ptms* | -0.516933955 | 0.01449546 | -0.66 | -0.66 |
| *Pxn* | -0.519325095 | 0.00862519 | -0.56 | -0.56 |
| *Arhgap21* | -0.520551542 | 0.02425255 | -0.87 | -0.87 |
| *Srf* | -0.523048901 | 0.01237515 | -0.94 | -0.96 |
| *Mknk2* | -0.525464258 | 0.02478351 | -0.69 | -0.69 |
| *Rplp2* | -0.526232118 | 0.04719447 | -0.9 | -0.92 |
| *Spns2* | -0.540136515 | 0.03385409 | -0.66 | -0.66 |
| *Ltbr* | -0.545952845 | 0.00250943 | -0.61 | -0.61 |
| *Cracr2b* | -0.554480345 | 0.03533255 | -0.67 | -0.72 |
| *Cdc42ep4* | -0.5560914 | 0.00652267 | -0.73 | -0.76 |
| *Elk4* | -0.558368281 | 0.01430232 | -0.78 | -0.82 |
| *Mgat4b* | -0.564252469 | 0.03851378 | -0.52 | -0.52 |
| *Creb3l2* | -0.567747136 | 0.03408738 | -0.72 | -0.72 |
| *Itpkc* | -0.568066287 | 0.01533145 | -0.64 | -0.77 |
| *Marveld1* | -0.569804924 | 0.02904874 | -0.68 | -0.75 |
| *Scpep1* | -0.573798335 | 0.00789459 | -0.77 | -0.77 |
| *Dtx3l* | -0.575650837 | 0.03161517 | -0.92 | -0.92 |
| *Klf3* | -0.578513175 | 0.00878709 | -0.77 | -0.92 |
| *Ralgds* | -0.580851627 | 0.01232029 | -0.68 | -0.68 |
| *Slc38a2* | -0.58447938 | 0.01728092 | -0.7 | -0.7 |
| *Mrpl14* | -0.5868317 | 0.03889297 | -0.59 | -0.59 |
| *Rgl1* | -0.590579195 | 0.02962139 | -1.05 | -1.05 |
| *Tnip2* | -0.594179563 | 0.02964859 | -0.59 | -0.59 |
| *Clic4* | -0.597415078 | 0.01501568 | -0.78 | -0.78 |
| *Hpcal1* | -0.599438236 | 0.00502412 | -0.52 | -0.52 |
| *Ing1* | -0.60322708 | 0.02627635 | -0.85 | -0.85 |

| | | | | |
|---|---|---|---|---|
| *Ppdpf* | -0.613937515 | 0.0050248 | -0.55 | -0.55 |
| *Blvrb* | -0.620820189 | 0.03948713 | -1.05 | -1.05 |
| *Tmcc3* | -0.624377999 | 0.00795486 | -0.92 | -0.92 |
| *Pros1* | -0.624644646 | 0.03017693 | -0.74 | -0.74 |
| *Ifngr1* | -0.627727721 | 0.00135363 | -1.02 | -1.04 |
| *Ppp1r15b* | -0.627765225 | 0.00859361 | -0.79 | -1.13 |
| *RGD1305464* | -0.628034034 | 0.03877064 | -0.65 | -0.65 |
| *F11r* | -0.631577738 | 0.00508848 | -0.69 | -0.69 |
| *Fam89b* | -0.633415118 | 0.00270271 | -0.72 | -0.74 |
| *Cpsf4* | -0.637912562 | 0.00234637 | -0.76 | -0.76 |
| *Pabpc1* | -0.643642087 | 0.01161343 | -1.08 | -1.08 |
| *Tor2a* | -0.647252704 | 0.01719035 | -1.28 | -1.28 |
| *Cars* | -0.65908069 | 0.03540092 | -0.65 | -0.65 |
| *Cst3* | -0.659332215 | 0.01902196 | -0.94 | -0.94 |
| *Tpm1* | -0.661082099 | 0.00748063 | -0.82 | -0.82 |
| *Acvr1b* | -0.662152407 | 0.01149969 | -0.73 | -0.94 |
| *Tspan2* | -0.662359245 | 0.01987439 | -0.73 | -0.78 |
| *Hist2h2ab* | -0.66261238 | 0.0361666 | -0.84 | -0.84 |
| *Aff1* | -0.664846287 | 0.01892007 | -0.69 | -0.69 |
| *Rps19* | -0.674624094 | 0.03630314 | -0.72 | -0.72 |
| *Mxd1* | -0.675660939 | 0.01398216 | -1 | -1 |
| *Tex264* | -0.679423807 | 0.01049146 | -0.67 | -0.67 |
| *Hmg20b* | -0.679851785 | 0.00175915 | -0.61 | -0.61 |
| *Itpr2* | -0.682583666 | 0.00054923 | -0.56 | -0.56 |
| *Tbc1d2b* | -0.684222228 | 0.00060975 | -0.86 | -0.86 |
| *Cd99* | -0.684267129 | 0.00345877 | -1.2 | -1.2 |
| *Plscr1* | -0.685800106 | 0.02037962 | -1.07 | -1.45 |
| *Ifih1* | -0.685898041 | 0.02329151 | -0.56 | -0.56 |
| *Rab30* | -0.69384775 | 0.04116045 | -0.7 | -0.7 |
| *LOC1003023[illegible]* | -0.693943739 | 0.04001126 | -0.56 | -0.56 |
| *Cmip* | -0.699038533 | 0.0222173 | -0.62 | -0.62 |
| *Ulk1* | -0.704659503 | 0.00426106 | -0.66 | -0.66 |
| *Scly* | -0.706400235 | 0.01881418 | -0.6 | -0.6 |
| *Zfyve21* | -0.706681641 | 0.01061357 | -0.78 | -0.78 |
| *Nr1d1* | -0.707993726 | 0.00550683 | -0.77 | -0.79 |
| *Plcg1* | -0.710058787 | 0.00074401 | -0.56 | -0.57 |
| *Psen2* | -0.710712427 | 0.00119865 | -0.56 | -0.56 |
| *Myo1c* | -0.717473057 | 0.00046939 | -0.73 | -0.79 |
| *Sat2* | -0.717601284 | 0.03288089 | -0.56 | -0.57 |
| *Clec14a* | -0.728867157 | 0.00863035 | -0.67 | -0.67 |
| *Aldh6a1* | -0.731929251 | 0.02590302 | -0.67 | -0.67 |
| *Pdlim5* | -0.733649269 | 0.00062064 | -0.46 | -0.46 |
| *H1f0* | -0.734956876 | 0.0059349 | -0.47 | -0.47 |
| *Sparc* | -0.735899187 | 0.0492313 | -0.72 | -0.72 |
| *Jak1* | -0.741957909 | 0.00100295 | -0.84 | -0.84 |

| | | | | |
|---|---|---|---|---|
| *Prickle3* | -0.743586892 | 0.02886727 | -0.74 | -0.77 |
| *Lurap1l* | -0.743809865 | 0.01832421 | -0.35 | -0.35 |
| *Klf6* | -0.746243756 | 0.01360338 | -0.9 | -0.9 |
| *Eif4ebp3* | -0.746374008 | 0.04335452 | -0.84 | -1 |
| *Shmt1* | -0.751334113 | 0.0153805 | -0.83 | -0.83 |
| *Extl3* | -0.755766391 | 0.00205542 | -0.72 | -0.72 |
| *RGD1311739* | -0.758049626 | 0.0153786 | -0.72 | -0.73 |
| *Eif2ak3* | -0.758334499 | 0.01449546 | -0.67 | -0.69 |
| *Midn* | -0.767728351 | 0.01363677 | -0.57 | -0.58 |
| *Grpel2* | -0.768971291 | 0.02131018 | -1.27 | -1.27 |
| *Cyp2t1* | -0.778583314 | 0.0353922 | -0.56 | -0.56 |
| *Plec* | -0.781557797 | 0.00714974 | -0.6 | -0.74 |
| *Sigirr* | -0.788309133 | 0.00094208 | -0.77 | -0.77 |
| *Asb13* | -0.788322206 | 0.03146392 | -0.76 | -0.76 |
| *RGD1309079* | -0.789932723 | 0.00156081 | -1.59 | -1.59 |
| *Cd34* | -0.792897918 | 0.00225654 | -0.88 | -0.94 |
| *Flna* | -0.80730852 | 0.03968244 | -0.81 | -0.81 |
| *Ung* | -0.808329881 | 0.00706854 | -1.02 | -1.02 |
| *Gas2l1* | -0.809580903 | 0.00452595 | -0.55 | -0.55 |
| *Dxo* | -0.820578057 | 0.01049146 | -0.79 | -0.79 |
| *Sorbs3* | -0.823437953 | 0.00350824 | -0.59 | -0.59 |
| *Tppp3* | -0.825679825 | 0.01837185 | -0.89 | -0.98 |
| *Ak3* | -0.826042673 | 0.00130352 | -0.72 | -0.87 |
| *Hectd2* | -0.826230536 | 0.01890945 | -0.54 | -0.54 |
| *RT1-T24-1* | -0.82748451 | 0.03594543 | -1.1 | -1.25 |
| *Arv1* | -0.829068799 | 0.02875322 | -0.57 | -0.58 |
| *Mast3* | -0.83018293 | 0.00452542 | -0.72 | -0.77 |
| *Crot* | -0.830866518 | 0.00202087 | -1.27 | -1.43 |
| *Cmtm8* | -0.837257274 | 0.01240048 | -0.52 | -0.52 |
| *Ifrd2* | -0.839599533 | 0.01318023 | -0.62 | -0.62 |
| *Nuak2* | -0.844436113 | 0.00856015 | -0.81 | -0.81 |
| *Egfl7* | -0.845096816 | 0.0021872 | -0.66 | -0.71 |
| *Dap* | -0.849157821 | 0.0092838 | -0.55 | -0.55 |
| *Prkch* | -0.849186209 | 0.01560548 | -0.87 | -0.88 |
| *Nudt4* | -0.85363613 | 0.00015237 | -0.88 | -0.88 |
| *Sypl1* | -0.855449655 | 5.19E-05 | -1.23 | -1.23 |
| *Cnksr3* | -0.856575171 | 0.00011903 | -0.7 | -0.82 |
| *Spry2* | -0.85974981 | 0.00293138 | -0.45 | -0.45 |
| *Gng11* | -0.861446849 | 0.00382348 | -0.84 | -0.88 |
| *Sidt2* | -0.865887738 | 1.09E-05 | -0.64 | -0.64 |
| *Plp2* | -0.870762519 | 2.22E-05 | -0.84 | -0.84 |
| *Rras* | -0.875009501 | 0.00124078 | -0.8 | -0.82 |
| *S100a10* | -0.875111148 | 0.00124078 | -0.69 | -0.78 |
| *Swap70* | -0.877489728 | 0.0304867 | -0.88 | -0.88 |
| *Erg* | -0.880039533 | 0.00874418 | -0.86 | -1.05 |

| | | | | |
|---|---|---|---|---|
| *Thbs3* | -0.880596073 | 0.01113843 | -1.05 | -1.05 |
| *Scrn2* | -0.881352315 | 0.01226883 | -0.69 | -0.69 |
| *Syde1* | -0.886897081 | 0.00571808 | -0.73 | -0.73 |
| *Dock9* | -0.894051322 | 2.65E-05 | -0.88 | -0.88 |
| *Wdtc1* | -0.900903994 | 0.00125535 | -0.51 | -0.51 |
| *Sat1* | -0.901252253 | 0.0073735 | -1.09 | -1.19 |
| *Trib1* | -0.907566448 | 2.62E-06 | -0.67 | -0.67 |
| *Gpt2* | -0.908604387 | 0.0166619 | -0.71 | -0.77 |
| *Ppp1r18* | -0.912740895 | 0.03946105 | -0.72 | -0.77 |
| *Sufu* | -0.913265734 | 0.04351321 | -0.77 | -0.77 |
| *Gpx1* | -0.917333477 | 0.00015877 | -0.76 | -0.76 |
| *Hspa1l* | -0.918145322 | 0.00294673 | -0.36 | -0.36 |
| *Foxa3* | -0.918145584 | 0.02373804 | -0.47 | -0.47 |
| *Clec2g* | -0.922140203 | 0.00026578 | -0.88 | -0.93 |
| *Slc1a5* | -0.924150818 | 0.00666647 | -0.82 | -0.82 |
| *Tpm2* | -0.933641621 | 0.03007938 | -0.7 | -0.7 |
| *Klf9* | -0.938070907 | 4.49E-05 | -0.7 | -0.7 |
| *Ehd4* | -0.940173206 | 9.42E-06 | -0.75 | -0.89 |
| *Ctsk* | -0.949695559 | 0.00824076 | -0.82 | -0.82 |
| *Pdlim4* | -0.95499139 | 0.00090287 | -0.52 | -0.52 |
| *Map7d1* | -0.955272941 | 0.00460056 | -0.57 | -0.58 |
| *Tjp2* | -0.955957672 | 1.11E-07 | -0.41 | -0.41 |
| *Sh3d21* | -0.962017505 | 0.02945868 | -0.64 | -0.64 |
| *Peak1* | -0.96430928 | 0.00178156 | -0.79 | -0.79 |
| *Gstm1* | -0.966453919 | 0.01377969 | -0.62 | -0.62 |
| *Aplf* | -0.966956339 | 0.0010105 | -0.72 | -0.72 |
| *Fam110a* | -0.968117419 | 0.00188619 | -0.67 | -0.67 |
| *Sfxn2* | -0.968582527 | 0.03910276 | -0.88 | -0.9 |
| *Bhlhe40* | -0.971051041 | 0.00072489 | -0.67 | -0.77 |
| *Echdc3* | -0.976513986 | 0.04565245 | -0.89 | -0.89 |
| *Ppp1r3c* | -0.98285857 | 0.00025702 | -1.01 | -1.01 |
| *Rnd3* | -0.983583938 | 3.15E-07 | -0.62 | -0.62 |
| *Il4r* | -0.984827237 | 0.01092893 | -0.48 | -0.48 |
| *Cd55* | -0.988407953 | 0.02458684 | -0.95 | -0.95 |
| *Jak3* | -0.988540951 | 0.00018655 | -0.86 | -0.86 |
| *Dctpp1* | -0.990925179 | 0.01190392 | -0.56 | -0.56 |
| *Stap2* | -0.996934247 | 0.01214516 | -0.54 | -0.54 |
| *Dlc1* | -1.000204161 | 0.00054923 | -0.81 | -0.81 |
| *Rps27l* | -1.003090709 | 0.00344574 | -0.77 | -0.77 |
| *Hist2h4* | -1.005837125 | 0.00010789 | -0.68 | -0.71 |
| *Acss3* | -1.009642362 | 0.00112917 | -0.72 | -0.72 |
| *Oaf* | -1.012975727 | 2.47E-06 | -0.68 | -0.68 |
| *Sdpr* | -1.019436772 | 0.00011787 | -1.05 | -1.05 |
| *Robo4* | -1.025779559 | 0.00074262 | -0.75 | -0.75 |
| *Bckdk* | -1.033536092 | 0.00303558 | -0.52 | -0.52 |

| | | | | |
|---|---|---|---|---|
| *Btg2* | -1.036059678 | 0.0003636 | -0.84 | -0.84 |
| *Cited2* | -1.039846891 | 5.03E-07 | -0.57 | -0.58 |
| *Bcl6* | -1.040165474 | 0.02101497 | -0.8 | -0.86 |
| *Parva* | -1.041276275 | 1.13E-07 | -0.52 | -0.52 |
| *A1cf* | -1.041843899 | 0.00870258 | -0.79 | -0.79 |
| *Ctsh* | -1.05228066 | 2.64E-05 | -0.43 | -0.43 |
| *Pik3c2b* | -1.052713822 | 0.00029704 | -0.6 | -0.6 |
| *Ccrl2* | -1.055710773 | 0.01510066 | -0.62 | -0.62 |
| *Jund* | -1.056547446 | 1.54E-06 | -0.73 | -0.83 |
| *Hic1* | -1.057102026 | 0.00215421 | -0.65 | -0.65 |
| *Bcl9l* | -1.062374869 | 0.00382632 | -0.51 | -0.51 |
| *Ehbp1l1* | -1.066566923 | 0.00156878 | -0.72 | -0.74 |
| *Bckdhb* | -1.067873591 | 0.00959061 | -0.88 | -0.88 |
| *LOC1009109[illegible]* | -1.068101304 | 0.00128481 | -0.8 | -0.81 |
| *Zmynd15* | -1.068940973 | 0.00896302 | -0.65 | -0.65 |
| *Ushbp1* | -1.070986196 | 0.00062434 | -0.57 | -0.58 |
| *Efna1* | -1.071367683 | 0.00038048 | -0.57 | -0.58 |
| *Nr1h3* | -1.071489778 | 0.00099658 | -0.56 | -0.57 |
| *Creg1* | -1.072644946 | 0.00017891 | -0.52 | -0.52 |
| *Irf1* | -1.074947664 | 0.01711041 | -0.95 | -1.04 |
| *Edem1* | -1.077818335 | 0.00017425 | -0.57 | -0.58 |
| *C1qtnf1* | -1.079144154 | 5.31E-05 | -0.6 | -0.6 |
| *Herpud1* | -1.083646128 | 0.00012509 | -0.67 | -0.69 |
| *Hes1* | -1.087010058 | 4.51E-06 | -0.62 | -0.62 |
| *Myrf* | -1.087246896 | 0.00171001 | -0.61 | -0.77 |
| *Foxo3* | -1.090617461 | 7.32E-08 | -0.75 | -0.75 |
| *Cd302* | -1.093050525 | 0.00677465 | -1.15 | -1.16 |
| *Rbms2* | -1.107335982 | 2.01E-06 | -0.83 | -0.83 |
| *Grasp* | -1.107953631 | 0.00055874 | -0.68 | -0.68 |
| *Ptafr* | -1.108390299 | 0.01536606 | -0.72 | -0.72 |
| *Notch4* | -1.109287106 | 0.00056812 | -0.7 | -0.7 |
| *Def6* | -1.117893084 | 0.03097577 | -0.65 | -0.65 |
| *Bach1* | -1.118184694 | 6.75E-07 | -0.64 | -0.64 |
| *Palmd* | -1.128554235 | 2.29E-05 | -0.8 | -0.8 |
| *Magi1* | -1.129066249 | 3.97E-06 | -0.43 | -0.43 |
| *Oplah* | -1.1303785 | 1.95E-06 | -0.53 | -0.53 |
| *Ier2* | -1.130954746 | 9.15E-06 | -0.79 | -0.79 |
| *Wfdc2* | -1.131368509 | 0.0002504 | -0.64 | -0.64 |
| *Rhbdf2* | -1.133227877 | 0.00038864 | -0.71 | -0.86 |
| *Epha1* | -1.136472693 | 0.00068136 | -0.78 | -0.78 |
| *Hist2h3c2* | -1.138625991 | 0.00198349 | -0.68 | -0.71 |
| *Pdgfa* | -1.139868651 | 1.54E-06 | -0.45 | -0.45 |
| *Creb3l1* | -1.142834147 | 0.00128126 | -0.63 | -0.63 |
| *Emp3* | -1.143100941 | 0.00045625 | -0.72 | -0.72 |
| *Mylip* | -1.143739534 | 9.40E-07 | -0.84 | -0.84 |

| | | | | |
|---|---|---|---|---|
| *Ppp1r15a* | -1.148442914 | 0.00050877 | -0.72 | -0.8 |
| *Nfil3* | -1.154633072 | 2.53E-06 | -0.66 | -0.86 |
| *Xbp1* | -1.159339333 | 0.00490494 | -0.69 | -0.84 |
| *RT1-T24-4* | -1.160391184 | 0.00103817 | -0.76 | -0.76 |
| *Mx2* | -1.162840842 | 0.00238129 | -0.53 | -0.53 |
| *Fkbp9* | -1.162952943 | 2.38E-10 | -0.54 | -0.54 |
| *Adcy4* | -1.16777101 | 0.00201998 | -0.81 | -0.81 |
| *Tmem209* | -1.174528538 | 0.00067878 | -0.81 | -0.81 |
| *Pycr1* | -1.175553896 | 0.02054619 | -0.82 | -0.82 |
| *Fzd4* | -1.184431959 | 0.00026032 | -0.58 | -0.63 |
| *Perp* | -1.184805061 | 5.05E-05 | -0.72 | -0.72 |
| *Axl* | -1.186955146 | 4.01E-05 | -0.71 | -0.71 |
| *Myadm* | -1.194350479 | 5.78E-06 | -0.85 | -0.85 |
| *Nfkbia* | -1.200498437 | 0.00047424 | -0.85 | -1.02 |
| *Gbp5* | -1.208356885 | 0.00557916 | -1.1 | -1.1 |
| *Sertad1* | -1.208787215 | 0.00075643 | -0.77 | -0.79 |
| *Slc28a2* | -1.217725326 | 0.00403673 | -0.75 | -0.75 |
| *Acer2* | -1.22142507 | 0.00010723 | -0.65 | -0.65 |
| *Jun* | -1.229126539 | 4.76E-06 | -0.95 | -0.95 |
| *Rapgef3* | -1.233597877 | 0.00022701 | -0.75 | -0.75 |
| *Usp2* | -1.236424208 | 0.01003703 | -0.78 | -0.78 |
| *Syngr2* | -1.237354708 | 1.60E-09 | -0.59 | -0.59 |
| *Rasip1* | -1.24196551 | 3.71E-06 | -0.74 | -0.74 |
| *Angpt2* | -1.244749157 | 0.00012075 | -0.95 | -0.95 |
| *Mpst* | -1.244916433 | 2.20E-06 | -0.52 | -0.54 |
| *Plekhg2* | -1.24702968 | 0.00292968 | -0.99 | -0.99 |
| *Clic3* | -1.249632443 | 0.02074039 | -0.53 | -0.53 |
| *Irf7* | -1.25314955 | 0.00292968 | -0.84 | -0.84 |
| *Mknk1* | -1.256455261 | 3.79E-05 | -0.61 | -0.61 |
| *Lamb2* | -1.258109792 | 9.03E-09 | -0.08 | 0.94 |
| *Cldn3* | -1.271874598 | 0.00035791 | -0.63 | -0.63 |
| *Psme3* | -1.274203234 | 1.37E-09 | -1.11 | -1.11 |
| *Stom* | -1.281683749 | 6.76E-05 | -1.25 | -1.25 |
| *Arhgap31* | -1.283902812 | 9.60E-05 | -0.84 | -0.84 |
| *Fcgrt* | -1.289625406 | 5.34E-11 | -0.74 | -0.74 |
| *Zc3h12a* | -1.291249397 | 0.00207382 | -0.47 | -0.47 |
| *LOC684871* | -1.291862258 | 9.33E-08 | -0.77 | -0.82 |
| *Arrb1* | -1.294609297 | 2.63E-07 | -0.62 | -0.62 |
| *Anxa1* | -1.294944627 | 9.93E-05 | -0.89 | -0.89 |
| *Ch25h* | -1.307818079 | 0.00088082 | -0.56 | -0.57 |
| *Sik1* | -1.309034007 | 2.01E-06 | -0.57 | -0.58 |
| *Olr1* | -1.311983933 | 0.03497708 | -0.87 | -0.87 |
| *Nrros* | -1.313779325 | 3.76E-05 | -1.02 | -1.02 |
| *Zfp503* | -1.313976995 | 7.76E-08 | -0.52 | -0.52 |
| *Junb* | -1.316509562 | 7.29E-05 | -0.76 | -0.76 |

| | | | | |
|---|---|---|---|---|
| *Evi2a* | -1.319060804 | 0.00193622 | -0.91 | -0.91 |
| *Lmo4* | -1.320037987 | 1.20E-07 | -0.73 | -0.85 |
| *Gbp2* | -1.338107089 | 0.00376063 | -1.02 | -1.02 |
| *Gucd1* | -1.34322789 | 8.13E-06 | -0.47 | -0.47 |
| *Rhoj* | -1.34617105 | 3.07E-07 | -0.84 | -0.84 |
| *Lipe* | -1.354067706 | 4.20E-06 | -0.8 | -0.8 |
| *Tgif1* | -1.35490131 | 1.03E-05 | -0.82 | -0.82 |
| *Cp* | -1.355824123 | 0.00034139 | -1.4 | -1.69 |
| *Il34* | -1.36481742 | 5.31E-05 | -0.77 | -0.77 |
| *Bcat2* | -1.366174095 | 0.00017425 | -0.82 | -0.88 |
| *Rnpepl1* | -1.366252669 | 9.37E-07 | -0.48 | -0.48 |
| *Bcl3* | -1.370147517 | 0.00382632 | -0.84 | -0.85 |
| *Klf4* | -1.388647186 | 0.00029146 | -0.49 | -0.49 |
| *Tnfsf10* | -1.389985427 | 1.69E-05 | -0.47 | -0.47 |
| *Gpr146* | -1.403531234 | 0.00099658 | -0.74 | -0.78 |
| *Fgl2* | -1.407862952 | 0.00335136 | -0.71 | -0.71 |
| *Mboat1* | -1.409877706 | 4.97E-07 | -0.39 | -0.39 |
| *Igfbp4* | -1.411968338 | 1.02E-10 | -0.66 | -0.66 |
| *Tmem79* | -1.414259075 | 0.00254748 | -0.69 | -0.69 |
| *Pim3* | -1.415647067 | 7.01E-10 | -0.61 | -0.61 |
| *Mgst2* | -1.418520474 | 3.40E-05 | -0.7 | -0.7 |
| *Ifrd1* | -1.418614869 | 5.51E-10 | -0.97 | -0.97 |
| *Itga5* | -1.420326973 | 7.42E-07 | -0.48 | -0.48 |
| *Bhlha15* | -1.422859547 | 0.00575085 | -0.48 | -0.48 |
| *Lfng* | -1.43220287 | 4.43E-11 | -0.67 | -0.67 |
| *Anxa2* | -1.439108233 | 8.53E-06 | -0.67 | -0.79 |
| *LOC1009105* | -1.439852082 | 1.69E-06 | -0.95 | -1.13 |
| *Gpr176* | -1.48063624 | 0.00252975 | -0.73 | -0.73 |
| *Reep5* | -1.48072949 | 2.62E-05 | -0.47 | -0.47 |
| *Fut2* | -1.485824925 | 0.00084957 | -0.77 | -0.77 |
| *Cyp27a1* | -1.490395562 | 8.16E-05 | -0.5 | -0.5 |
| *Akr1cl* | -1.50508889 | 0.0008723 | -1.48 | -1.48 |
| *Mgat4a* | -1.517677166 | 0.00041505 | -0.93 | -0.93 |
| *Nfib* | -1.532297934 | 1.07E-09 | -0.95 | -1.13 |
| *Ephx1* | -1.540012781 | 5.66E-09 | -0.47 | -0.47 |
| *Plxnd1* | -1.551977782 | 4.67E-09 | -0.66 | -0.66 |
| *Ptgir* | -1.565343232 | 0.00056337 | -0.53 | -0.53 |
| *Ahnak* | -1.565960535 | 0.00105749 | -1.06 | -1.06 |
| *Piezo1* | -1.586419475 | 8.14E-07 | -0.8 | -0.8 |
| *Kif5a* | -1.593576394 | 0.00473464 | -0.86 | -0.86 |
| *Pla2g12a* | -1.593914413 | 1.18E-05 | -0.42 | -0.42 |
| *Msra* | -1.599664596 | 0.00061984 | -0.81 | -0.81 |
| *Ddit4* | -1.62475095 | 6.15E-12 | -0.6 | -0.6 |
| *Slc25a35* | -1.645772335 | 0.00236449 | -0.56 | -0.57 |
| *Dgat2* | -1.657228818 | 4.41E-07 | -0.73 | -0.73 |

| | | | | |
|---|---|---|---|---|
| *Errfi1* | -1.660167082 | 8.79E-08 | -0.67 | -0.77 |
| *Zfp36l1* | -1.688270945 | 3.21E-09 | -0.66 | -0.81 |
| *Metrnl* | -1.696315869 | 3.70E-07 | -0.77 | -0.77 |
| *Nfkbiz* | -1.708731779 | 4.43E-06 | -1.02 | -1.02 |
| *Ndrg2* | -1.712241284 | 1.06E-09 | -0.47 | -0.47 |
| *Adrb2* | -1.714781893 | 0.00245537 | -0.73 | -0.89 |
| *ST7* | -1.715247045 | 1.07E-06 | -0.43 | -0.43 |
| *Fabp4* | -1.72602357 | 0.0002386 | -0.77 | -0.77 |
| *Ier3* | -1.735733471 | 1.03E-05 | -0.81 | -0.81 |
| *Pmp22* | -1.754321312 | 7.07E-12 | -0.5 | -0.5 |
| *Socs3* | -1.765289037 | 3.98E-05 | -0.71 | -0.78 |
| *Ltbp4* | -1.796793239 | 2.61E-09 | -0.9 | -0.9 |
| *Snx33* | -1.822166797 | 6.28E-19 | -0.43 | -0.43 |
| *Plekha4* | -1.841907457 | 1.27E-07 | -0.72 | -0.8 |
| *Csrnp1* | -1.843362632 | 4.63E-07 | -0.59 | -0.59 |
| *Parp16* | -1.861930019 | 1.12E-06 | -0.41 | -0.41 |
| *Sel1l* | -1.864057215 | 9.29E-05 | -0.95 | -0.95 |
| *Pparg* | -1.8807179 | 0.00039487 | -0.47 | -0.47 |
| *Magix* | -1.906008621 | 0.00054628 | -0.83 | -0.83 |
| *Dram1* | -1.916697854 | 5.29E-07 | -0.71 | -0.76 |
| *RGD1305928* | -1.929397118 | 0.02479784 | -1.12 | -1.35 |
| *Rnh1* | -1.96791872 | 1.32E-07 | -0.56 | -0.56 |
| *Ppard* | -1.971175032 | 7.32E-11 | -0.74 | -0.74 |
| *Zfp36* | -1.972051542 | 4.68E-08 | -0.96 | -0.99 |
| *Nat8b* | -1.973867904 | 3.54E-05 | -0.73 | -0.77 |
| *Fam109b* | -2.054086095 | 1.25E-05 | -0.77 | -0.77 |
| *Trpm4* | -2.061647297 | 1.10E-11 | -0.51 | -0.51 |
| *Tnf* | -2.101881623 | 0.00201979 | -0.95 | -0.95 |
| *Fut1* | -2.126850993 | 9.40E-07 | -0.56 | -0.56 |
| *Slc1a3* | -2.135732156 | 3.60E-12 | -1.06 | -1.06 |
| *Procr* | -2.266468169 | 2.69E-08 | -0.67 | -0.67 |
| *Ssfa2* | -2.275706611 | 1.32E-14 | -0.45 | -0.45 |
| *Metrn* | -2.379856791 | 8.93E-10 | -1 | -1 |
| *Gda* | -2.388879765 | 3.65E-11 | -0.69 | -0.69 |
| *Cdc42ep1* | -2.392560796 | 6.23E-15 | -0.49 | -0.49 |
| *Mpz* | -2.493595543 | 0.00267129 | -0.56 | -0.56 |
| *Cxcl9* | -2.546864549 | 1.07E-05 | -0.28 | -1.2 |
| *Myc* | -2.557702609 | 1.13E-16 | -0.56 | -0.56 |
| *Cebpe* | -2.57810204 | 0.0265603 | -0.78 | -0.78 |
| *Cish* | -2.649561686 | 1.32E-17 | -0.73 | -0.73 |
| *Mir27a* | -2.73729527 | 0.01740591 | -0.68 | -0.68 |
| *Gamt* | -2.772215531 | 1.25E-09 | -0.87 | -0.87 |
| *Mt1* | -2.800206934 | 1.57E-11 | -0.79 | -0.79 |
| *Adamts4* | -2.875268325 | 3.50E-09 | -0.22 | 0.76 |
| *Slc16a7* | -2.914826182 | 1.29E-11 | -0.88 | -0.88 |

Supplemental Figure S1. No direct association between levels of normalized gene expression and genomic location of histone marks

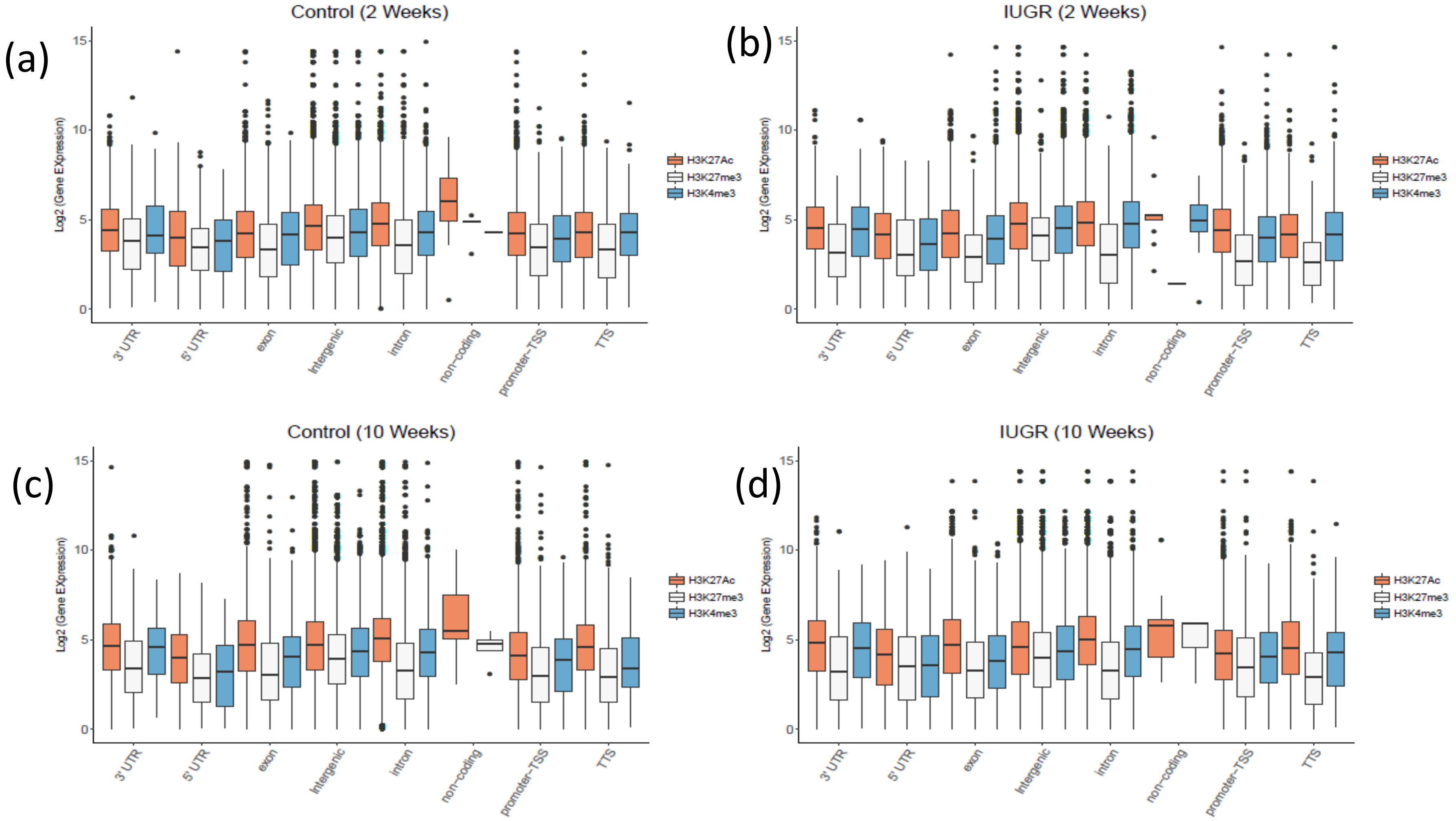


**Supplemental Figure S1:** No direct association between levels of normalized gene expression and genomic location of histone marks. (a) and (b) represent normalized gene expression for histone marks at different genomic locations in 2-wk control and IUGR islets, respectively. (c) and (d) represent normalized gene expression for histone marks at different genomic locations in 10-wk control and IUGR islets, respectively. Red bars represent H3K27Ac, white bars represent H3K27me3, and blue bars represent H3K4me3.

Supplemental Figure S2 Genome browser snapshots for examples of genes potentially regulated by all three histone modifications

(a) Tfam

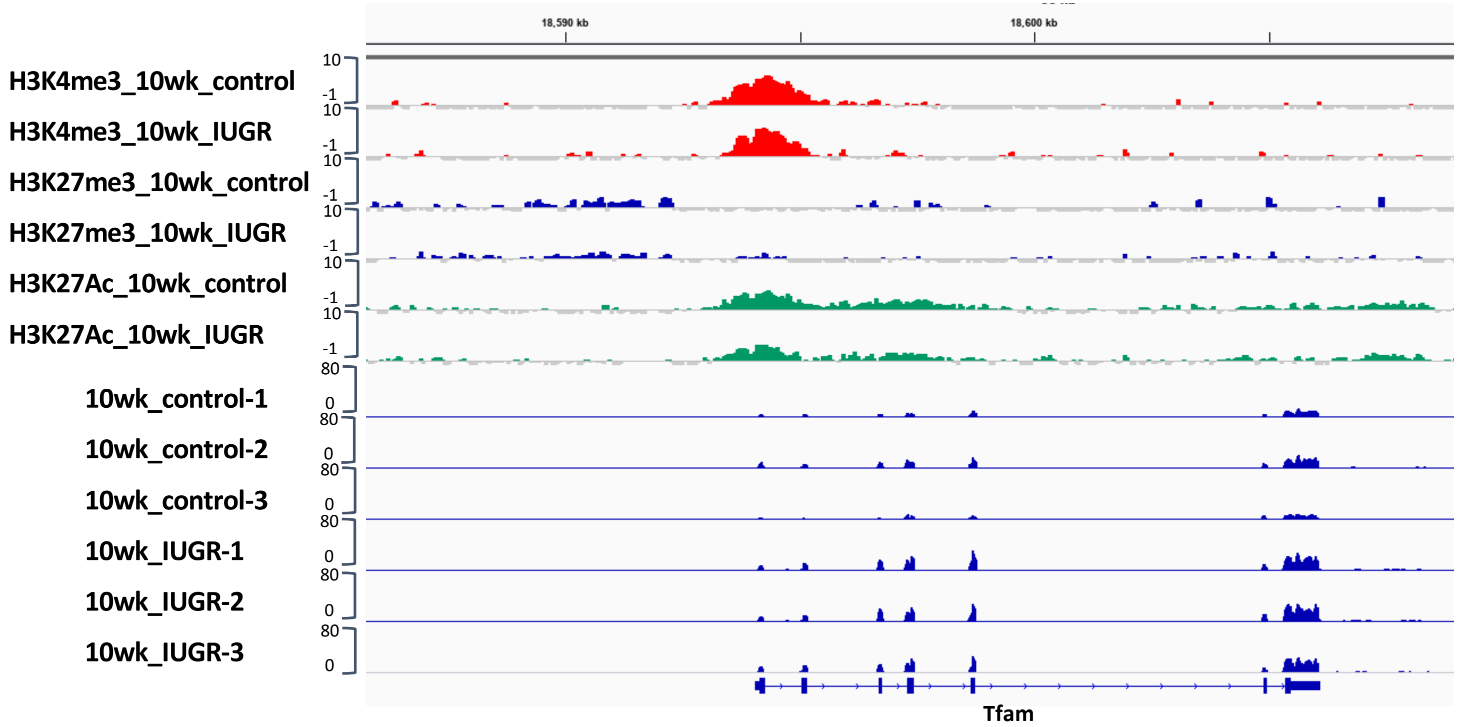


**Supplemental Figure S2:** Genome browser snapshots showing examples of genes potentially regulated by all 3 histone modifications. (a) Tfam gene and its vicinity region. Red color represents ChIP-seq track for H3K4me3 mark. Blue color represents ChIP-seq track for H3K27me3 mark. Green color represents ChIP-seq track for H3K27Ac mark. The level of gene expression is shown in the bottom with RNA-seq track.

Supplemental Figure S2 Genome browser snapshots for examples of genes potentially regulated by all three histone modifications

(b) Mcf2l

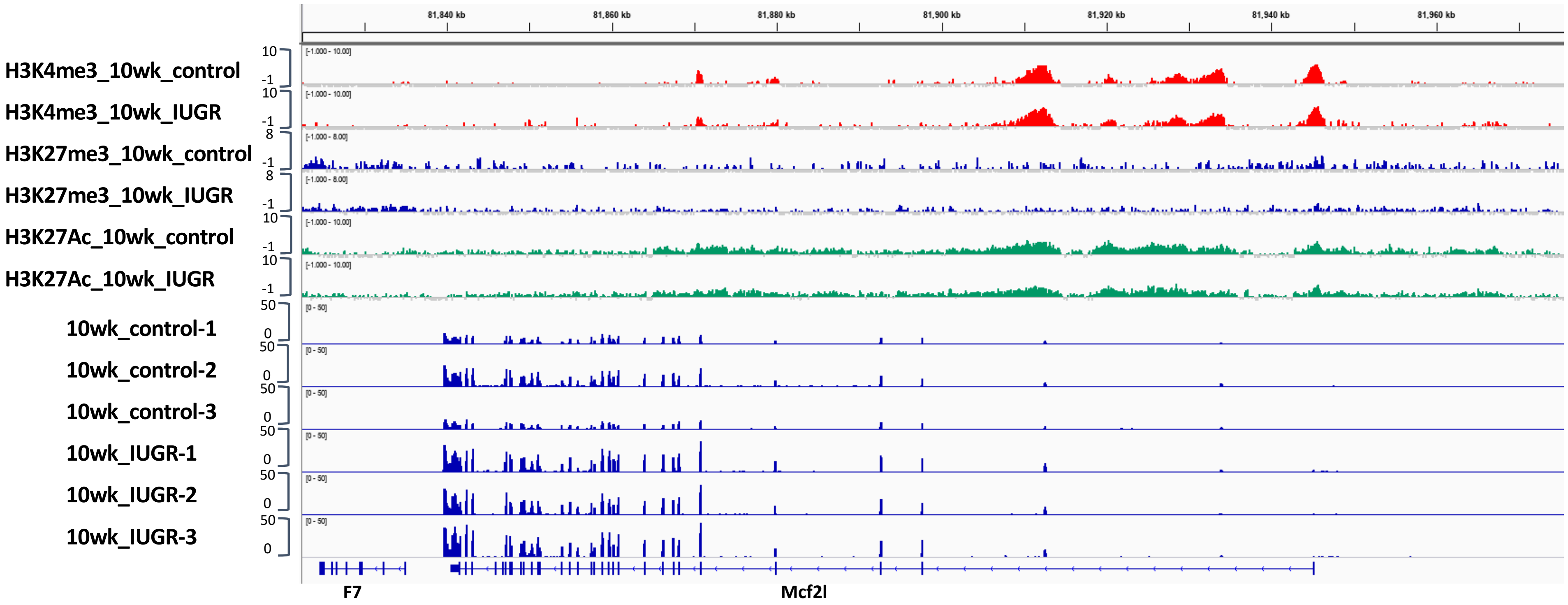


**Supplemental Figure S2:** Genome browser snapshots showing examples of genes potentially regulated by all 3 histone modifications. (b) Mcf2l gene and its vicinity region. Red color represents ChIP-seq track for H3K4me3 mark. Blue color represents ChIP-seq track for H3K27me3 mark. Green color represents ChIP-seq track for H3K27Ac mark. The level of gene expression is shown in the bottom with RNA-seq track.

Supplemental Figure S2 Genome browser snapshots for examples of genes potentially regulated by all three histone modifications

(c) Slc28a2

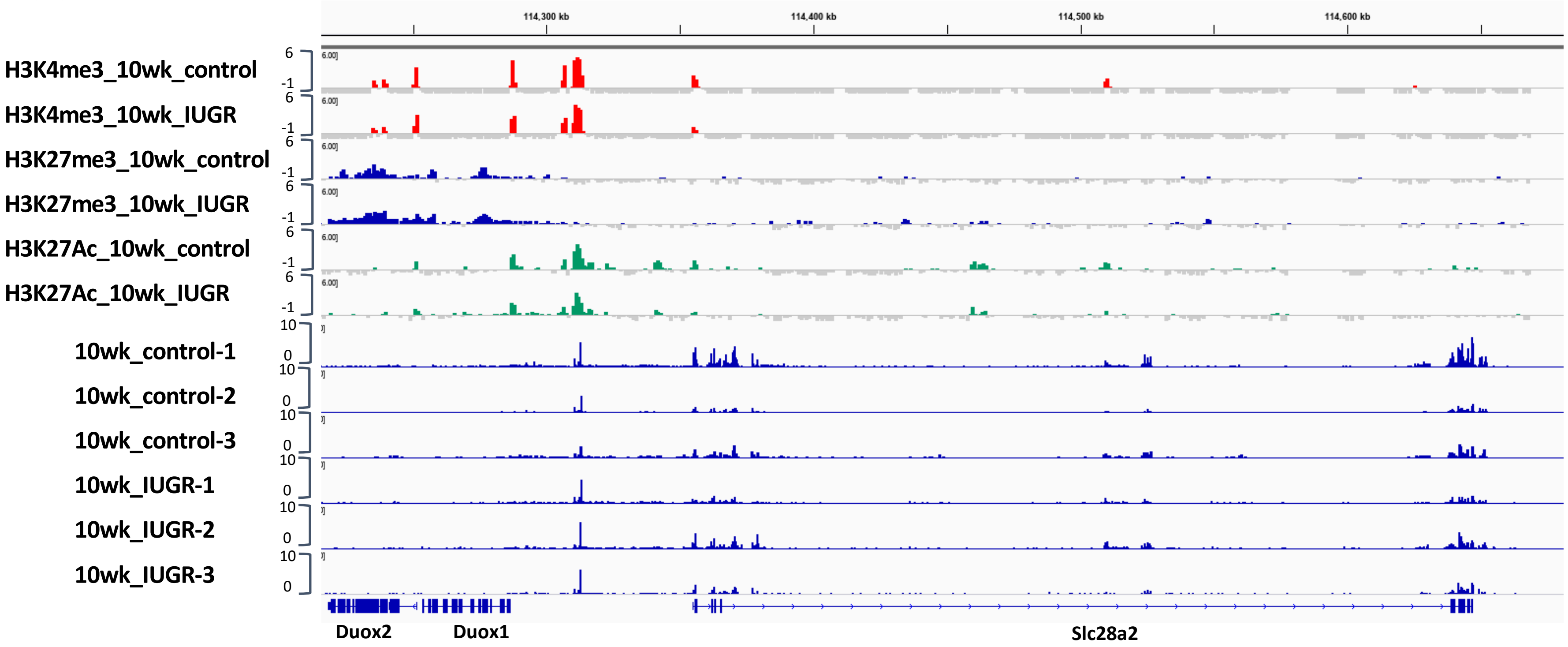


**Supplemental Figure S2:** Genome browser snapshots showing examples of genes potentially regulated by all 3 histone modifications. (c) Slc28a2 genes and its vicinity region. Red color represents ChIP-seq track for H3K4me3 mark. Blue color represents ChIP-seq track for H3K27me3 mark. Green color represents ChIP-seq track for H3K27Ac mark. The level of gene expression is shown in the bottom with RNA-seq track.

Supplemental Figure S3 Genome browser snapshots for examples of potential bivalent genes

(a) Fgf21

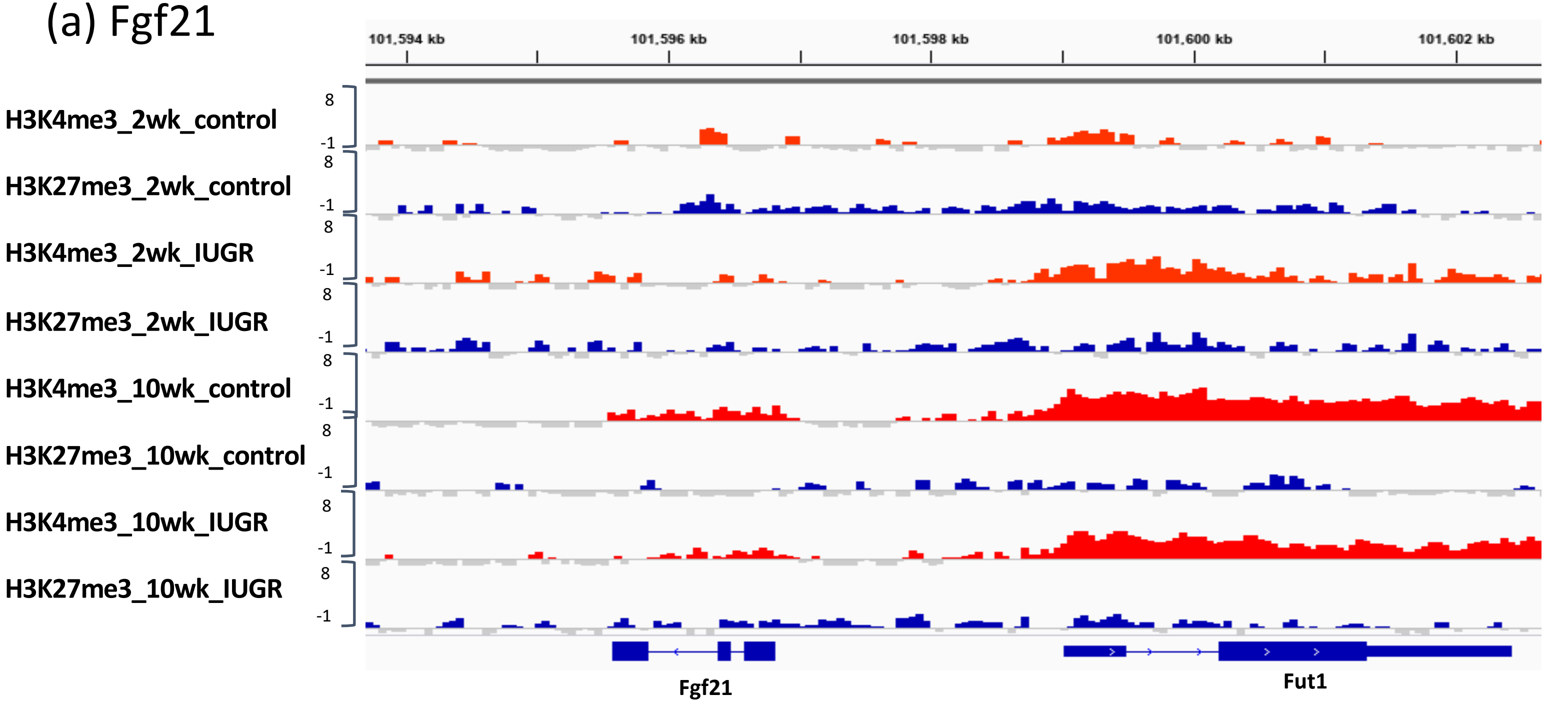


**Supplemental Figure S3:** Genome browser snapshots showing examples of potential bivalent genes. (a) Fgf21gene and its vicinity region. Red color represents ChIP-seq track for H3K4me3 mark. Blue color represents ChIP-seq track for H3K27me3 mark.

Supplemental Figure S3 Genome browser snapshots for examples of potential bivalent genes

(b) Acod1

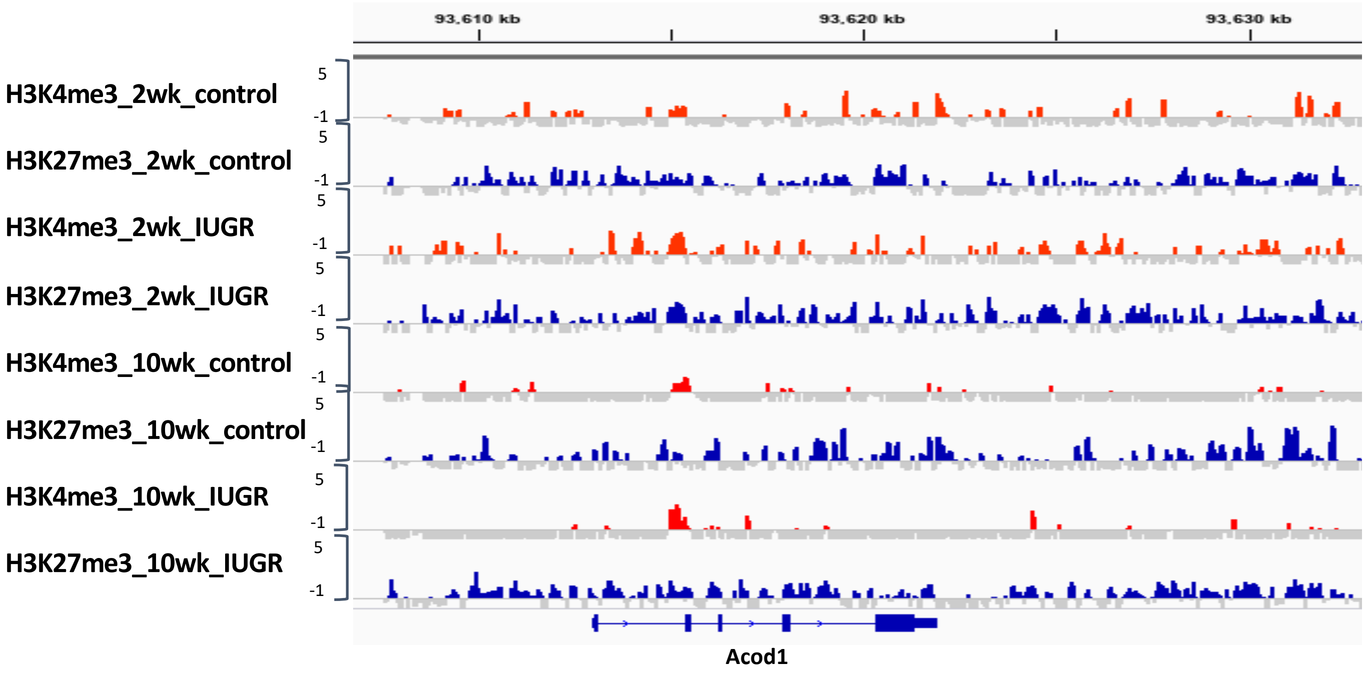


**Supplemental Figure S3:** Genome browser snapshots showing examples of potential bivalent genes. (b) Acod1 gene and its vicinity region. Red color represents ChIP-seq track for H3K4me3 mark. Blue color represents ChIP-seq track for H3K27me3 mark.
